# MBD3/NuRD Facilitates Induction of Pluripotency in a Context-Dependent Manner

**DOI:** 10.1016/j.stem.2014.04.019

**Published:** 2014-07-03

**Authors:** Rodrigo L. dos Santos, Luca Tosti, Aliaksandra Radzisheuskaya, Isabel M. Caballero, Keisuke Kaji, Brian Hendrich, José C.R. Silva

**Affiliations:** 1 Wellcome Trust – Medical Research Council Cambridge Stem Cell Institute and Department of Biochemistry, University of Cambridge, Tennis Court Road, Cambridge CB2 1QR, UK; 2 Doctoral Programme in Experimental Biology and Biomedicine, Centre for Neuroscience and Cell Biology and Institute for Interdisciplinary Research, University of Coimbra, 3030-789 Coimbra, Portugal; 3 MRC Centre for Regenerative Medicine, University of Edinburgh, Edinburgh BioQuarter, 5 Little France Drive, Edinburgh EH16 4UU, UK

## Abstract

The Nucleosome Remodeling and Deacetylase (NuRD) complex is essential for embryonic development and pluripotent stem cell differentiation. In this study, we investigated whether NuRD is also involved in the reverse biological process of induction of pluripotency in neural stem cells. By knocking out MBD3, an essential scaffold subunit of the NuRD complex, at different time points in reprogramming, we found that efficient formation of reprogramming intermediates and induced pluripotent stem cells from neural stem cells requires NuRD activity. We also show that reprogramming of epiblast-derived stem cells to naive pluripotency requires NuRD complex function and that increased MBD3/NuRD levels can enhance reprogramming efficiency when coexpressed with the reprogramming factor NANOG. Our results therefore show that the MBD3/NuRD complex plays a key role in reprogramming in certain contexts and that a chromatin complex required for cell differentiation can also promote reversion back to a naive pluripotent cell state.

## Highlights

• Mbd3 facilitates the initiation of neural stem cell reprogramming
• Mbd3 is also required for efficient iPSC generation from EpiSCs and preiPSCs
• Overexpression of Mbd3/NuRD facilitates reprogramming in a context-dependent manner

## Graphical Abstract

**Figure:**
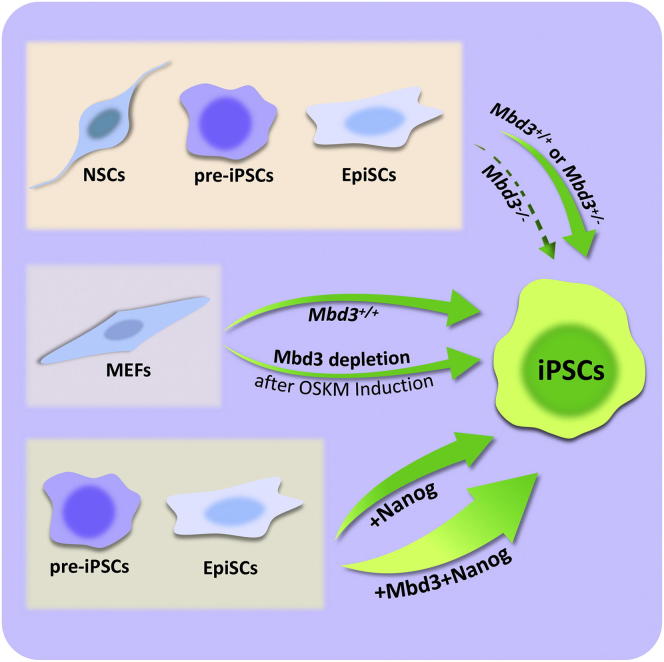

## Teaser

dos Santos et al. show that Mbd3/NuRD plays a positive role in reprogramming in certain contexts and that overexpression of Mbd3 facilitates Nanog-mediated reprogramming.

## Introduction

Reprogramming of somatic cells to naive pluripotency can be robustly driven by the combined action of transcription factors and culture cues. Among the reprogramming transcription factors, OCT4 plays a central role, as it is sufficient and essential for the induction of pluripotent cells (Kim et al., 2009; Radzisheuskaya et al., 2013; Radzisheuskaya and Silva, 2014). OCT4 interactome studies in embryonic stem cells (ESCs) revealed members of the Nucleosome Remodeling and Deacetylase (NuRD) complex as its highest confidence interactors (Ding et al., 2012; Liang et al., 2008; Pardo et al., 2010; van den Berg et al., 2010). NuRD is composed of six core subunits with at least two enzymatic activities involved in gene regulation: histone deacetylase activity of HDAC1/2 subunits and ATP-dependent chromatin remodeling activity of Mi-2a/β subunits (Lai and Wade, 2011; McDonel et al., 2009). Methyl-CpG binding domain protein 3 (MBD3) is an essential scaffold protein of the NuRD complex, in the absence of which the complex is not assembled (Kaji et al., 2006; Zhang et al., 1999). Embryos lacking MBD3 die shortly after implantation (Hendrich et al., 2001; Kaji et al., 2007) and *Mbd3*-null ESCs are viable but show severely impaired lineage commitment and exhibit limited differentiation capacity (Kaji et al., 2006; Reynolds et al., 2012a, 2012b). Chromatin remodeling plays an important role in reprogramming to naive pluripotency (Apostolou and Hochedlinger, 2013; Papp and Plath, 2013). Because the NuRD complex is a high confidence interactor of Oct4 and a key regulator of developmental cell state transitions, we have investigated its involvement in the induction of pluripotency.

## Results

### MBD3 Facilitates the Initiation of Reprogramming from Neural Stem Cells

**Figure 1:**
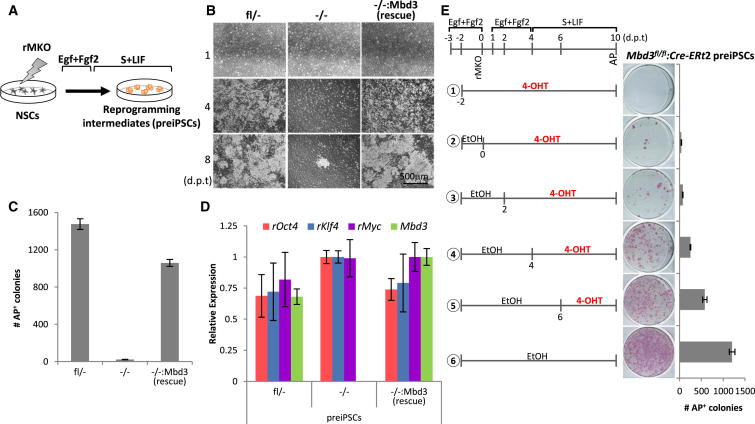
MBD3 Facilitates the Initiation of Reprogramming (A) Experimental design used to address the kinetics and efficiency of initiation of reprogramming in NSCs with different *Mbd3* genotypes. NSCs were transduced with retroviruses encoding *cMyc*, *Klf4*, and *Oct4* (rMKO), maintained in Egf+Fgf2 medium for 3 days, and then switched to S+LIF medium. (B) Phase images of the reprogramming intermediates (preiPSCs) emerging from *Mbd3*^*fl/−*^, *Mbd3*^*−/−*^, and *Mbd3*^*−/−*^*:Mbd3* (rescue) NSCs at different days posttransduction (d.p.t.). (C) Efficiency of preiPSC colony formation per 2.5 × 10^5^ NSCs as assessed by alkaline phosphatase (AP) staining at day 9 posttransduction. (D) qRT-PCR analysis of retroviral transgenes (*rOct4*, *rKlf4*, and *rMyc*) and *Mbd3* expression in the obtained preiPSCs maintained in S+LIF. Three independent NSCs transductions were carried out and gene expression was assessed 12 days after transduction. Values are normalized to *Gapdh* value and shown as relative to the highest value. (E) Time course of MBD3 requirement during preiPSC formation. *Mbd3*^*fl/fl*^ NSCs were stably transfected with pCAG-CreERt2 transgene, transduced with retroviral transgenes, and treated with 4-OHT at indicated time points to induce Cre-mediated deletion of the floxed alleles during reprogramming. Ethanol (EtOH) was used as a control. The encircled numbers correspond to different conditions. PreiPSC colony formation was assessed by AP staining at day 10 posttransduction and is presented as the number of colonies per 7.5 × 10^4^ NSCs. The error bars indicate STDEV.

To address the requirement of the NuRD complex in the reprogramming process, we established an *Mbd3*^*−/−*^ clonal neural stem cell (NSC) line from *Mbd3*^*fl/−*^ NSCs and an *Mbd3*^*−/−*^ rescue NSC line (*Mbd3*^*−/−*^*:Mbd3*) by stable transfection of an *Mbd3* transgene (Figures S1A–S1C available online). These NSC lines were transduced with retroviruses encoding *cMyc*, *Klf4*, and *Oct4* (rMKO) to initiate their reprogramming and were then switched to serum plus LIF (S+LIF) conditions (Figure 1A), which typically results in the formation of highly proliferative reprogramming intermediates, or preiPSCs (Silva et al., 2008). When we used retroviruses encoding *GFP* (rGFP), equal percentages of GFP^+^ cells were observed 72 hr after transduction of *Mbd3*^*fl/−*^ or *Mbd3*^*−/−*^ NSCs, indicating that *Mbd3* deletion does not affect transduction efficiency (Figures S1D and S1E). Strikingly, the kinetics of preiPSC emergence was markedly delayed in the *Mbd3*^*−/−*^ cells. While *Mbd3*-expressing preiPSCs dominated the culture by day 4 posttransduction (d.p.t.), *Mbd3*^*−/−*^ preiPSCs emerged only by 7–8 d.p.t. (Figure 1B). In addition, the number of emerging alkaline-phosphatase-positive (AP^+^) *Mbd3*^*−/−*^ preiPSC colonies was significantly reduced compared to parental and rescue cell lines (Figure 1C and Figure S1F). Nevertheless, it was possible to establish and expand *Mbd3*^*−/−*^ preiPSCs, although less efficiently and with delayed kinetics. Both *Mbd3*^*−/−*^ NSCs and *Mbd3*-null preiPSCs derived from them exhibited slower proliferation, consistent with previous reports of *Mbd3*^*−/−*^ ESCs (Kaji et al., 2006; Sims and Wade, 2011) (Figures S1G and S1H). *Mbd3*^*−/−*^ preiPSCs expressed slightly higher levels of retroviral transgenes compared to control cells (Figure 1D), suggesting that dosage of reprogramming factors is not the reason for the reduced efficiency of reprogramming initiation that we observed.

To further dissect the requirement for MBD3 in the initiation of reprogramming, we analyzed the effect of *Mbd3* deletion at different experimental time points. For this experiment, we stably transfected *Mbd3*^*fl/fl*^ NSCs with *Cre-ERt2*, which enabled Cre-mediated excision of the floxed *Mbd3* alleles upon addition of 4-hydroxytamoxifen (4-OHT) (Figures S1I–S1L). We found that earlier removal of *Mbd3* reduced the number of preiPSC colonies formed (Figure 1E). We also obtained similar results after conditional deletion of *Mbd3* exon 1 (ex1fl) which removes all but a small amount of a truncated MBD3 protein isoform (MBD3C) (Aguilera et al., 2011; Kaji et al., 2006) (Figures S1M and S1N).

Taken together, these results demonstrate that lack of a functional NuRD complex strongly impairs the initiation of reprogramming from NSCs.

### MBD3 Is Required for Efficient iPSC Generation from NSCs, preiPSCs, and EpiSCs

**Figure 2:**
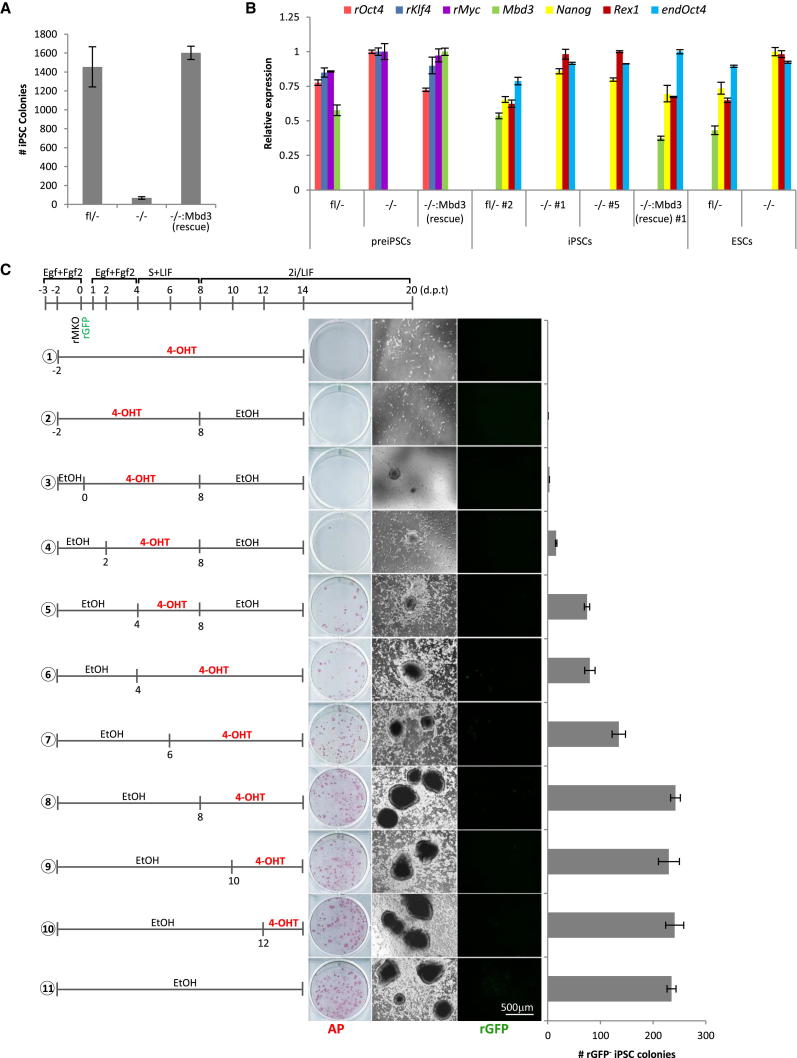
MBD3 Is Required for Efficient iPSC Generation (A) Quantification of iPSC colonies generated from *Mbd3*^*fl/−*^, *Mbd3*^*−/−*^, and *Mbd3*^*−/−*^*:Mbd3* (rescue) preiPSCs after 2i/LIF culture for 12 days. Colony number is per 1.0 × 10^5^ preiPSCs. (B) qRT-PCR analysis of retroviral transgenes, *Mbd3*, and pluripotency-associated factors in preiPSCs and corresponding derived iPSCs. qRT-PCR values are normalized to *Gapdh* value and shown as relative to the highest value. (C) *Mbd3*^*fl/fl*^*:Cre-ERt2* NSCs were transduced with rMKO and rGFP, maintained in Egf+Fgf2 medium for 3 days, switched to S+LIF for 4 more days to allow preiPSC emergence, and then switched to 2i/LIF conditions to induce iPSC formation. 4-OHT was added at different time points (before or after preiPSC emergence) to induce *Mbd3*-floxed alleles excision. The encircled numbers correspond to different conditions. At day 20 after transfection, GFP^−^ iPSC colonies were counted and subsequently stained for AP. The number of colonies is presented per 7.5 × 10^4^ NSCs. The error bars indicate STDEV.

We then evaluated the role of MBD3 in later stages of reprogramming. To induce completion of the reprogramming process, *Mbd3*^*fl/−*^, *Mbd3*^*−/−*^, and rescued *Mbd3*^*−/−*^*:Mbd3* preiPSCs were switched to serum-free medium containing LIF and inhibitors of both mitogen-activated protein kinase and glycogen synthase kinase-3 signaling (2i/LIF) (Silva et al., 2008), and the resulting iPSC colonies were scored 12 days later. We observed that the efficiency of conversion to naive pluripotency of *Mbd3*^*−/−*^ preiPSCs is strongly reduced compared to *Mbd3*^*fl/−*^ and *Mbd3*^*−/−*^*:Mbd3* preiPSCs (Figure 2A). The *Mbd3*^*fl/−*^, *Mbd3*^*−/−*^, and *Mbd3*^*−/−*^*:Mbd3* iPSCs that were obtained could be expanded clonally in 2i/LIF, and they exhibited reactivation of the pluripotency transcriptional program and silencing of the retroviral reprogramming transgenes as expected (Figure 2B). The *Mbd3*^*−/−*^ iPSCs were phenotypically similar to previously reported *Mbd3*-null ESCs (Kaji et al., 2006), exhibiting impaired embryoid body differentiation and slower proliferation (Figures S2A and S2B). We also observed that *Mbd3* deletion in an established preiPSC line before the 2i/LIF medium switch impaired reprogramming to naive pluripotency (Figures S2C and S2D).

Next we performed a time course experiment to define the time frame during reprogramming for which MBD3 is required. For this analysis, we transduced *Mbd3*^*fl/fl*^*:Cre-ERt2* NSCs with rMKO and rGFP and treated them with 4-OHT at different experimental time points (Figure 2C). The growth medium was changed to S+LIF 4 days after transduction and subsequently, 4 days later, to 2i/LIF. The number of iPSC colonies exhibiting silencing of retroviral GFP expression was assessed 12 days after 2i/LIF medium switch (Figure 2C and Figure S2E). We observed that the number of iPSC colonies formed was proportional to the amount of time cells expressed MBD3 during the initiation phase of reprogramming (prior to 2i/LIF culture). We observed neither a reduction nor a gain of reprogramming efficiency when *Mbd3* was deleted at the 2i/LIF stage. Regardless of the stage of *Mbd3* deletion, the resulting iPSCs displayed a pluripotency-associated transcriptional signature (Figure S2F). Thus, our data suggest that MBD3 is specifically required for the initiation and intermediate stage of NSC reprogramming rather than establishment of pluripotency.

**Figure 3:**
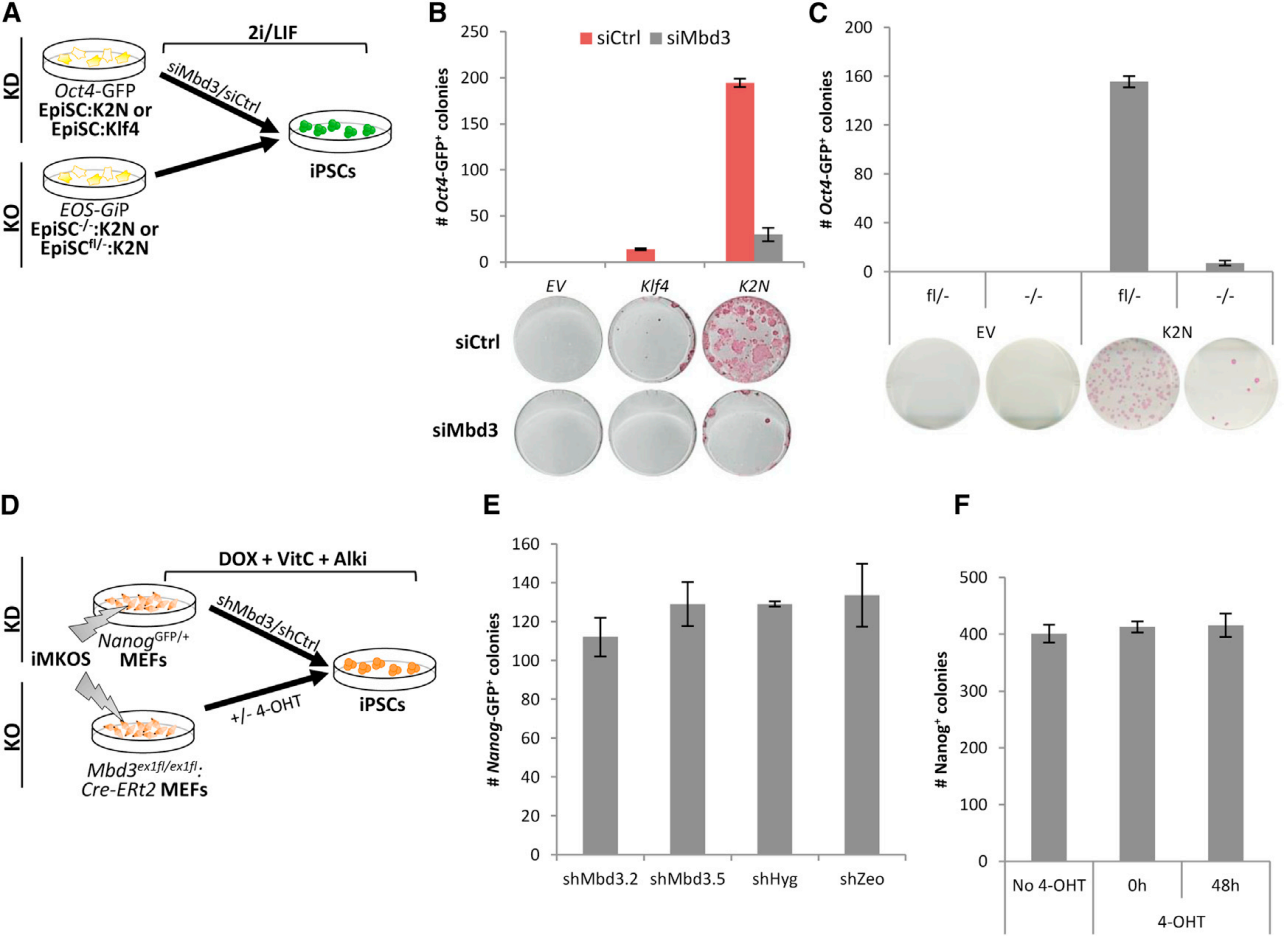
Requirement of MBD3 in Other Reprogramming Systems (A) Experimental designs used to analyze the effect of *Mbd3* KD and KO on EpiSC reprogramming efficiency. For the KD experiments, wild-type EpiSCs (carrying an *Oct4*-GFP cassette), stably transfected with pPB-CAG-Klf2.2A.Nanog (K2N) or pPB-CAG-Klf4, were transfected with either siMbd3 or siControl (siCtrl) and, after 24 hr, were plated in 2i/LIF for 12 days. For the KO experiments, *Mbd3*^*−/−*^ or *Mbd3*^*fl−*^ EpiSCs carrying an *Oct4*-GFP reporter (*EOS*-GiP), stably transfected with K2N (or empty vector control, EV), were plated in 2i/LIF for 12 days. (B and C) The efficiency of EpiSC reprogramming after *Mbd3* removal, either by KD (B) or KO (C), was assessed by counting *Oct4*-GFP^+^ colonies. Representative AP stained plates are also indicated. 1.0 × 10^4^ EpiSCs were plated in (B). 1.5 × 10^4^ EpiSCs were plated in (C). (D) Experimental designs used to analyze the effect of *Mbd3* KD and *Mbd3* exon 1 KO. For the KD experiments, *Nanog*-GFP MEFs transfected with doxycycline-inducible MKOS piggyBac transposon (iMKOS) were cultured in S+LIF + DOX + vitamin C (vitC) + Alki 24 hr before lentiviral infections of shMbd3 or shControls. For the KO experiments, *Mbd3*^*ex1fl/ex1fl*^ MEFs transfected with iMKOS were infected with pMX-Cre-ERt2. Reprogramming was carried out in S+LIF + DOX + vitC + Alki, and 4-OHT was added either at the time of DOX administration (0h) or 48 hr later (48h). (E) Number of *Nanog*-GFP^+^ iPSC colonies at day 13 of reprogramming upon infection of indicated shRNAs. (F) Number of Nanog^+^ colony numbers determined by immunofluorescence after 13 days of reprogramming of *Mbd3*^*ex1fl/ex1fl*^*:Cre*-ERt2 MEFs. The error bars indicate STDEV. Typical iMKOS positive cell number at day 2 of reprogramming is 1.0–3.0 × 10^4^ cells per well, providing 1%–2% reprogramming efficiency.

Epiblast stem cells (EpiSCs) can be reprogrammed to naive pluripotency by a combination of overexpression of at least one transcription factor, such as KLF4, KLF2, or NANOG, and the use of serum-free 2i/LIF medium, which not only promotes reprogramming of EpiSCs but also blocks their self-renewal (Guo et al., 2009; Silva et al., 2009). To examine the role of MBD3 in reprogramming in this context, we stably transfected wild-type EpiSCs carrying an *Oct4-*GFP reporter with piggyBac (PB) vectors constitutively expressing *Klf2* and *Nanog* (K2N) or *Klf4*, and we then transfected these with either small interfering RNA (siRNA) against *Mbd3* or control siRNA (Figure 3A). Strikingly, *Mbd3* knockdown led to a complete impairment of KLF4-mediated reprogramming and to a 6-fold reduction in the reprogramming ability of K2N (Figure 3B and Figures S3A and S3B). Similar results were obtained when EpiSCs with *Mbd3* genetic knockout were used (Figure 3C and Figures S3C and S3D).

All the results described above indicate that MBD3 is critical for efficient reprogramming in the contexts that we examined, contrasting with previous reports (Luo et al., 2013; Rais et al., 2013). To examine whether this difference is a reflection of the specific reprogramming systems that we used, we performed PB-mediated reprogramming of mouse embryonic fibroblasts (MEFs) combined with *Mbd3* depletion using two different approaches (Figure 3D). First, we used an *Mbd3* knockdown system in which *Nanog*-GFP MEFs were treated with doxycycline (DOX) for the induction of the MKOS or STEMCCA reprogramming cassettes (Kaji et al., 2009; Sommer et al., 2009) and cultured in S+LIF medium supplemented with vitamin C and Alki (Tgfβ signaling inhibitor). Twenty-four hours after induction they were transduced with lentiviruses expressing shRNA against *Mbd3* (Figure 3E and Figures S3E–S3G). Second, we depleted *Mbd3* by treating *Cre-ERt2*-transduced *Mbd3*^*ex1fl/ex1fl*^ MEFs with 4-OHT at 0 hr or 48 hrs after induction of reprogramming factor expression (Figure 3F and Figures S3H and S3I). While both systems demonstrated about 80% downregulation of MBD3 protein, neither impacted on the efficiency of MEF reprogramming. However, depletion of MBD3 protein would take a few days from the time of 4-OHT administration, so it is possible that in this system cells go through the most critical stage of reprogramming with MBD3 protein still present at a sufficient level.

From our experiments we therefore found that, depending on the reprogramming context, MBD3/NuRD depletion can either have no apparent effect on reprogramming or significantly impair the transition to naive pluripotency.

### Overexpression of MBD3/NuRD Can Facilitate Reprogramming

**Figure 4:**
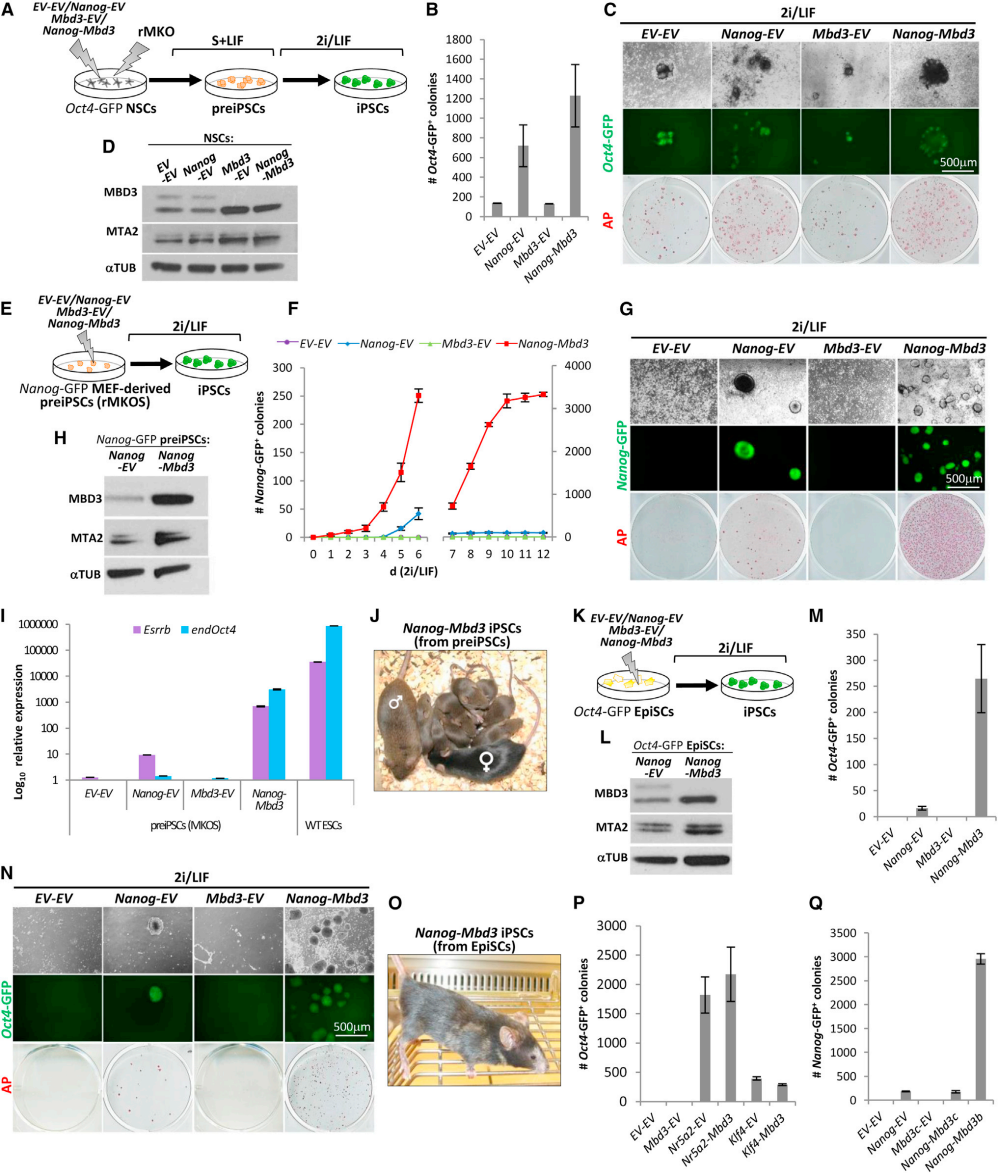
Overexpression of MBD3/NuRD Facilitates NANOG-Mediated Reprogramming (A) Experimental design used to address the effect of MBD3 overexpression on NSC reprogramming. NSCs carrying an *Oct4*-GFP cassette were stably transfected with pPB-CAG-Nanog and pPB-CAG-Mbd3b or pPB-CAG-empty controls, transduced with rOKM, cultured in Egf+Fgf2 medium for 3 days, switched to S+LIF medium for 6 days, and then switched to 2i/LIF conditions. (B) Quantification of *Oct4*-GFP^+^ colonies after 12 days in 2i/LIF conditions. Colony number is per 1.0 × 10^5^ NSCs. (C) Phase and GFP images and AP staining of the iPSCs obtained from NSCs overexpressing respective transgenes. (D) Western blot analysis of MBD3, MTA2, and TUBULIN (TUB) protein expression in NSCs overexpressing the indicated transgene combinations. (E) Experimental design used to address the effect of MBD3/NuRD overexpression on the conversion of preiPSCs to iPSCs. PreiPSCs (carrying a *Nanog*-GFP) were stably transfected with the same transgene combinations as in (A) and plated in 2i/LIF conditions for 12 days. (F) The kinetics of the emergence of *Nanog*-GFP^+^ colonies from the transgenic preiPSCs during a 12 day culture in 2i/LIF conditions (y axis scale changes at day 7). Colony number is per 1.0 × 10^5^ preiPSCs. (G) Phase and GFP images and AP staining of the iPSCs formed from preiPSCs overexpressing respective transgenes. (H) Western blot analysis of MBD3, MTA2, and TUBULIN (TUB) protein expression in preiPSCs overexpressing NANOG or NANOG and MBD3. (I) qRT-PCR analysis of *Esrrβ* and endogenous (end) *Oct4* expression in preiPSCs 12 days after stable transgene transfection and culture in S+LIF (y axes in log_10_ scale). The expression levels of *Esrrβ* and endogenous *Oct4* in these *Nanog-Mbd3* preiPSCs are 5% and 3%, respectively, of the expression levels of WT ESCs in 2i/LIF. The *Esrrβ* expression level is also approximately 80 times greater than that of *Nanog-EV* preiPSCs and 700 times greater than that of *Mbd3-EV* and *EV-EV* preiPSCs. The endogenous *Oct4* expression level is approximately 2,000 times greater than that of *Nanog-EV* preiPSCs and 3,000 times greater than that of *Mbd3-EV* and *EV-EV* preiPSCs. qRT-PCR values are normalized to *Gapdh* value and shown as relative to the highest value. (J) Germ line contribution of *Nanog-Mbd3* iPSCs generated from preiPSCs (brown color). Cells were treated with TAT-Cre for reprogramming transgene excision prior to blastocyst injection. Chimeric father, C57BL/6 mother, and pups resulting from cross can be viewed. (K) Experimental design used to address the effect of MBD3/NuRD overexpression on EpiSC reprogramming. EpiSCs (carrying an *Oct4*-GFP) were stably transfected with the same transgene combinations as in (A) and (E) and plated in 2i/LIF conditions for 12 days. (L) Western blot analysis of MBD3, MTA2, and TUBULIN (TUB) protein expression in EpiSCs overexpressing NANOG or NANOG and MBD3. (M) Quantification of *Oct4*-GFP^+^ colonies after 12 days of 2i/LIF culture. Colony numbers are per 2.0 × 10^4^ EpiSCs. (N) Phase and GFP images and AP staining of the iPSCs formed from EpiSCs overexpressing respective transgenes. (O) Chimera of *Nanog-Mbd3* iPSCs generated from EpiSCs (brown color). Cells were treated with TAT-Cre for reprogramming transgene excision prior to blastocyst injection. (P) Quantification of *Oct4*-GFP^+^ colonies after 12 days of 2i/LIF culture, generated from EpiSCs transfected with *Klf4* or *Nr5a2* (together or not with *Mbd3*). Colony numbers are per 2.0 × 10^4^ EpiSCs. (Q) Quantification of *Nanog*-GFP^+^ colonies after 12 days of 2i/LIF culture generated from MEF-derived preiPSCs stably transfected with *Nanog* alone, or *Nanog* together with *Mbd3b* or *Mbd3c*. Colony numbers are per 1.0 × 10^5^ preiPSCs. The error bars indicate STDEV.

Because the complete removal of *Mbd3* or a decrease in its expression can significantly impair the generation of both preiPSCs and iPSCs from NSCs, and iPSCs from preiPSCs and EpiSCs, we tested whether MBD3 levels are limiting for reprogramming. For that, *Oct4*-GFP reporter NSCs and *Nanog*-GFP MEF-derived preiPSCs were stably transfected with *Mbd3* (Figures 4A and 4E). MBD3 overexpression had neither a positive nor detrimental effect on the efficiency of iPSC formation in both systems (Figures 4B, 4C, 4F and 4G). However, combined overexpression of MBD3 and NANOG in MEF-derived preiPSCs led to accelerated reprogramming kinetics and an up to 30-fold increase in reprogramming efficiency compared to *Nanog*-*Empty* vector (*EV*) control (Figures 4F and 4G and Figures S4A and S4B). This synergistic effect correlated with the upregulation of both *Esrrβ* and endogenous *Oct4* expression, to 5% and 3% of the expression levels of wild-type ESCs, respectively, prior to induction of pluripotency by 2i/LIF medium switch (Figure 4I). Interestingly, MBD3 overexpression in these cell lines caused an increase in protein levels of MTA2, a core subunit of the NuRD complex degraded in the absence of MBD3 (Figures 4D and 4H) (Kaji et al., 2006). This suggests that the effects of MBD3 overexpression are potentially attributable to the total amount, and subsequently total activity, of the NuRD complex. We also found that EpiSCs overexpressing both Nanog and MBD3 showed a 30-fold increase in the ability to generate iPSCs relative to a *Nanog* only control (Figures 4K–4N). Importantly, all iPSCs generated by the overexpression of NANOG and MBD3 exhibited the molecular properties expected for naive pluripotent cells (Figures S4C–S4F) as well as chimera and germline competence after the excision of reprogramming transgenes (Figures 4J and 4O). We did not see reprogramming synergy with the NuRD complex for two other known reprogramming factors, KLF4 and NR5A2 (Figure 4P and Figure S4G).

The MBD3 isoform that we used for the rescue and overexpression experiments was MBD3B, the most abundant isoform by protein levels in ESCs (Figure S4K). In contrast to MBD3B, we found that MBD3C did not synergize with NANOG (Figure 4Q and Figures S4H–S4J). The two isoforms differ in only the first 60 N-terminal amino acids (Figure S4H), indicating that this region is of importance for NANOG-dependent MBD3 ability to facilitate reprogramming.

These results demonstrate that MBD3 overexpression does not impair induction of naive pluripotency and that it can in fact facilitate reprogramming in conjunction with enhanced NANOG expression.

## Discussion

In this study we have identified a positive facilitator role for MBD3/NuRD in transcription-factor-mediated reprogramming of NSCs and EpiSCs. In our analyses, we found that genetic or siRNA-mediated depletion of *Mbd3* led to a reduction in the efficiency of reprogramming in these contexts, but not in reprogramming of MEFs. More specifically, we found through time course experiments that MBD3/NuRD function is particularly important during the initiation phase of reprogramming of NSCs and is more dispensable in the later stages when the pluripotency network is becoming more stably established.

We also found that MBD3 overexpression, with resulting higher levels of the NuRD complex, facilitates reprogramming of MEF-derived preiPSCs and EpiSCs when combined with expression of NANOG, but not with other tested reprogramming factors, which is consistent with previous observations that NuRD complex subunits are high confidence protein interactors of both OCT4 and NANOG (Costa et al., 2013; Ding et al., 2012; Gagliardi et al., 2013; Liang et al., 2008; Pardo et al., 2010; van den Berg et al., 2010). In our experiments the N teminus of the MBD3B isoform, which has previously been suggested to be required for protein-protein interactions (Aguilera et al., 2011), appeared to be required for the observed synergistic effect with NANOG in reprogramming (Figure 4Q). In the future we will aim to understand how NANOG and MBD3 work together to drive cells (preiPSCs) that are arrested in the reprogramming process toward pluripotency.

Our data suggest that the NuRD complex might be facilitating gene activation during reprogramming. Interestingly, MBD3 was recently shown to localize to the regulatory sequences of active genes (Günther et al., 2013; Reynolds et al., 2013; Shimbo et al., 2013), including ESC super-enhancers (Hnisz et al., 2013). Moreover, genome-wide expression analysis revealed that 61% of differentially expressed genes are downregulated after *Mbd3* deletion in ESCs (Reynolds et al., 2012b). Although some of this decrease in transcription might be due to indirect effects, it seems likely that the NuRD complex acts at enhancers as a mediator of transcription-factor-induced gene activation and thus could also interact with pluripotency factors such as NANOG to support genome-wide reprogramming. In addition, NuRD has been proposed to mediate transient mTOR downregulation and subsequent activation of autophagy, a key step during early stages of reprogramming (Wang et al., 2013).

Our results are in apparent disagreement with two recent reports that suggested an inhibitory role for MBD3 in reprogramming (Luo et al., 2013; Rais et al., 2013), including one (Rais et al., 2013) that argued that reduction or deletion of *Mbd3* leads to rapid deterministic reprogramming with 100% efficiency. There are a number of differences between our study and these two previous reports, including the choice of reprogramming cassettes and the reprogramming culture conditions. In contrast to our study, Rais et al. (2013) used a secondary system for somatic cell reprogramming and lentiviral cassette delivery, and it has been reported that both of these factors influence iPSC generation efficiency (Stadtfeld and Hochedlinger, 2010). Moreover, distinct reprogramming factor stoichiometry can provide varying intracellular environments, which may show different dependencies on MBD3 activity for reprogramming. In addition, in our hands heterozygous *Mbd3*^*fl/−*^ ESCs express MBD3 at nearly wild-type levels (Figures S4K–S4M; Reynolds et al., 2012b), but Rais et al. (2013) reported that their *Mbd3*^*fl/−*^ ESCs expressed MBD3 at 20% of wild-type levels. Further examination of these and other practical and procedural differences between our study and the previous work should help clarify the basis of the apparent differences seen.

Overall, taking into account the results that we report here and previous studies, our conclusion is that at least in some contexts MBD3/NuRD plays a positive role in reprogramming, and that loss of MBD3 expression leads to a reduction in the efficiency of the reprogramming process.

NuRD plays well-documented roles in controlling gene expression and developmental transitions in a wide variety of different metazoan systems (Ahringer, 2000; McDonel et al., 2009; Reynolds et al., 2013). MBD3 is known to be required for embryonic development and pluripotent cell differentiation (Kaji et al., 2006, 2007), and the composition of the complex or specific interactions of its individual subunits may regulate different aspects of its function (Allen et al., 2013; Reynolds et al., 2013). Further insights into the function of the NuRD complex during different cell state transitions will help us understand the process of induced pluripotency as well as embryonic development.

## Experimental Procedures

### Cell Culture

Platinum-E, preiPSCs, and MEFs were cultured in GMEM (Sigma-Aldrich) supplemented with 10% FCS, 1× NEAA, 1× Pen/Strep, 1 mM sodium pyruvate, 0.1 mM 2-mercaptoethanol, 2 mM L-glutamine, and 20 ng/ml of LIF (homemade), indicated as *S*+LIF medium throughout. ESCs and iPSCs were maintained in N2B27-based medium (DMEM/F12 and Neurobasal [both Life Technologies] in 1:1 ratio, 1× Pen/Strep, 0.1 mM 2-mercaptoethanol, 2 mM L-glutamine, 1:200 N2 [PAA], and 1:100 B27 [Life Technologies]) supplemented 20 ng/ml of LIF and 2i inhibitors: CHIR99021 (3 μM) and PD0325901 (1 μM), indicated as 2i/LIF throughout (Ying et al., 2008). NSCs were cultured in DMEM/F12 (GIBCO) supplemented with 1× NEAA, 0.1 mM 2-mercaptoethanol, 1× Pen/Strep, 1:100 B27, 1:200 N2 supplement, 4.5 μM HEPES, 0.03 M glucose, 120 μg/ml BSA, 10 ng/ml of Egf (Peprotech), and 20 ng/ml of Fgf2 (homemade), indicated as Egf+Fgf2 medium throughout. EpiSCs were maintained in N2B27-based medium containing 12 ng/ml of Fgf2 and 20 ng/ml of Activin A (homemade), indicated as Fgf2/Act.A medium throughout. EpiSCs and NSCs were cultured on plastic coated with fibronectin (10 μg/ml, Millipore) or laminin (10 μg/ml, Sigma-Aldrich), respectively. All other cell types were grown on gelatine. All cell types were maintained at 7% CO_2_. For Cre-mediated transgene excision, cells were treated with 500 nM of 4-OHT.

### Derivation of Cell Lines

#### NSCs

Brains from *Mbd3*^*fl/fl*^ and *Mbd3*^*ex1fl/ex1fl*^ E13.5 embryos were dissected, dissociated in Egf+Fgf2 medium, and plated onto the laminin-coated cell culture flasks. *Mbd3*^*fl/−*^ NSCs were derived from ESCs as described (Pollard et al., 2006). Briefly, ESCs were seeded on gelatinized 10 cm dishes in N2B27 medium for 7 days. After this period, cells were trypsinized and plated on nongelatinized dishes for 3 days in Egf+Fgf2 medium. The emergent neurospheres were then seeded on gelatinized plates and maintained in monolayer in Egf+Fgf2 medium. For Cre-excision of the *Mbd3* floxed allele, *Mbd3*^*fl*/−^ NSCs were nucleofected with a pCAG-Cre-ires-Puro plasmid and clonal lines of *Mbd3*^*−/−*^ NSCs were expanded.

#### MEFs

Organ-deprived carcasses from E12.5 or E13.5 embryos were dissociated into small pieces, trypsinized, and plated in S+LIF medium.

#### EpiSCs

*Mbd3*^*fl/−*^ and *Mbd3*^*−/−*^ EpiSCs were derived from ESCs as previously described (Guo et al., 2009). Briefly, ESCs transfected with pPB-EOS-GFP-ires-Puro (*EOS*-GiP; GFPiresPuro under the control of early transposon promoter and *Oct4* and *Sox2* enhancers) were cultured in Fgf2/Act.A medium for at least 10 passages before analysis. To obtain a pure EpiSC culture, GFP^+^ cells were removed by FACS.

## Supplemental Information

Document S1. Figures S1–S4 and Supplemental Experimental Procedures

Document S2. Article plus Supplemental Information
