# MBD3/NuRD Facilitates Induction of Pluripotency in a Context-Dependent Manner

**DOI:** 10.1016/j.stem.2014.08.005

**Published:** 2014-09-04

**Authors:** Rodrigo L. dos Santos, Luca Tosti, Aliaksandra Radzisheuskaya, Isabel M. Caballero, Keisuke Kaji, Brian Hendrich, José C.R. Silva

(Cell Stem Cell *15*, 102–110; July 3, 2014)

A reader brought to our attention the fact that the presentation of Figure S3A was inappropriate because presented lanes were positioned adjacent to each other for the figure without an indication that they were not organized in that way on the original blot. During investigation of this concern, we also noticed that there was an error in one of the tubulin lanes presented. In this experiment, we looked at the effect of individual siRNAs, pooled administration of four together, and, as discussed in the text, used the combined approach in subsequent experiments. The original version of the figure only presented data for the effect of the pooled siRNAs, so to maximize clarity we have now corrected Figure S3A to show the entire blot for both the 48 hr and 72 hr time points. The supplemental information file for the paper has been adjusted to reflect this change. We apologize for any confusion caused by the original presentation of the figure.

