# Supplementary material for: MBD3/NuRD Facilitates Induction of Pluripotency in a Context-Dependent Manner
Source: Cell Stem Cell. 2014 Jul 3;15(1):102–10. doi: 10.1016/j.stem.2014.04.019 (PMC4082719; doi:10.1016/j.stem.2014.04.019)
Supplement: Document S1. Figures S1–S4 and Supplemental Experimental Procedures [file mmc1.pdf]

**Cell Stem Cell, Volume *15***

**Supplemental Information**

# MBD3/NuRD Facilitates Induction of Pluripotency in a Context-Dependent Manner

**Rodrigo L. dos Santos, Luca Tosti, Aliaksandra Radzisheuskaya, Isabel M. Caballero, Keisuke Kaji, Brian Hendrich, and José C. R. Silva**

# Supplemental Figures 1-4

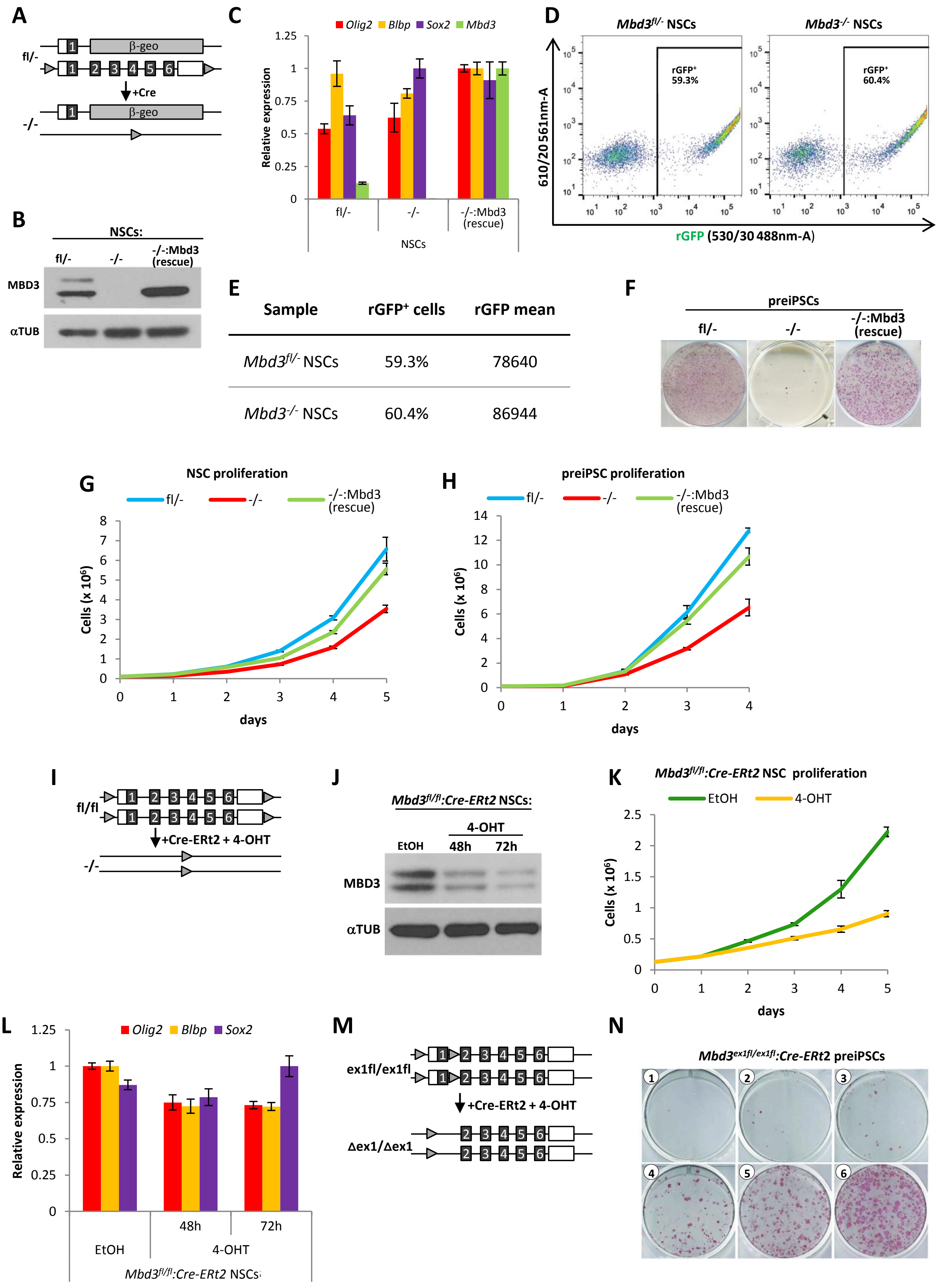

**Figure S1, related to Figure 1 - Mbd3 facilitates the initiation of reprogramming.**

**(A)** Schematic representation of *Mbd3* loci in NSCs $Mbd3^{fl/-}$ and $Mbd3^{-/-}$. Exons are indicated as dark grey boxes, non-coding sequences are indicated as unfilled boxes, and light grey triangles represent loxP sites. **(B)** Western blot analysis of MBD3 and α-TUBULIN (αTUB) protein levels in the $Mbd3^{fl/-}$, $Mbd3^{-/-}$ and $Mbd3^{-/-}$:*Mbd3* (rescue) NSCs used for reprogramming experiments in Figures 1A-C. **(C)** qRT-PCR analysis of NSC markers (*Sox2*, *Olig2*, *Blbp*) and *Mbd3* expression levels in NSCs. **(D)** Retroviral GFP (rGFP) expression 72h after transduction of $Mbd3^{fl/-}$ or $Mbd3^{-/-}$ NSCs with pMX-GFP, assessed by flow cytometry. $GFP^+$ gates are shown. **(E)** Table indicates the percentage of $rGFP^+$ cells and rGFP mean 72h after transduction of $Mbd3^{fl/-}$ or $Mbd3^{-/-}$ NSCs with pMX-GFP. **(F)** Alkaline phosphatase (AP) staining of preiPSCs 9 days post-transduction of NSCs $Mbd3^{fl/-}$, $Mbd3^{-/-}$ and rescue NSC lines. **(G)** Cell proliferation analysis of $Mbd3^{fl/-}$, $Mbd3^{-/-}$ and rescue NSC lines. **(H)** Cell proliferation analysis of $Mbd3^{fl/-}$, $Mbd3^{-/-}$ and $Mbd3^{-/-}$:*Mbd3* preiPSCs lines. **(I)** Schematic representation of *Mbd3* loci in $Mbd3^{fl/fl}$ NSCs before and after transfection with the pCAG-CreERt2 transgene and treatment with 4-OHT. Exons are indicated as dark grey boxes, non-coding sequences are indicated as unfilled boxes, and light grey triangles represent loxP sites. **(J)** Western blot analysis of MBD3 and α-TUBULIN (αTUB) protein levels in the $Mbd3^{fl/fl}$:*Cre-ERt2* NSCs treated with tamoxifen (4-OHT) or ethanol (EtOH). **(K)** Cell proliferation analysis of NSCs $Mbd3^{fl/fl}$:*Cre-ERt2*, in the presence of 4-OHT or EtOH. **(L)** qRT-PCR analysis of NSC markers expression in $Mbd3^{fl/fl}$:*Cre-ERt2* NSCs treated with 4-OHT or EtOH. qRT-PCR values are normalized to *Gapdh* value and shown as relative to the highest value. The error bars indicate STDEV. **(M)** Schematic representation of *Mbd3* loci in $Mbd3^{ex1fl/ex1fl}$ NSCs before and after transfection with pCAG-Cre-ERt2 transgene and treatment with 4-OHT. Exons are indicated as dark grey boxes, non-coding sequences are indicated as unfilled boxes, and

light grey triangles represent loxP sites. **(N)** Time-course of the removal of *Mbd3* exon 1 during initiation of reprogramming. *Mbd3*$^{ex1fl/ex1fl}$ NSCs were stably transfected with the pCAG-Cre-ERt2 transgene, transduced with retroviruses expressing reprogramming factors and treated with 4-OHT at different reprogramming time points to induce Cre-mediated deletion of the floxed alleles. EtOH was used as a control. The efficiency of preiPSC formation was assessed by AP staining at day 10 post transduction. The encircled numbers correspond to different conditions. For schematic representation of different conditions and understanding of labelling, refer to Figure 1E.

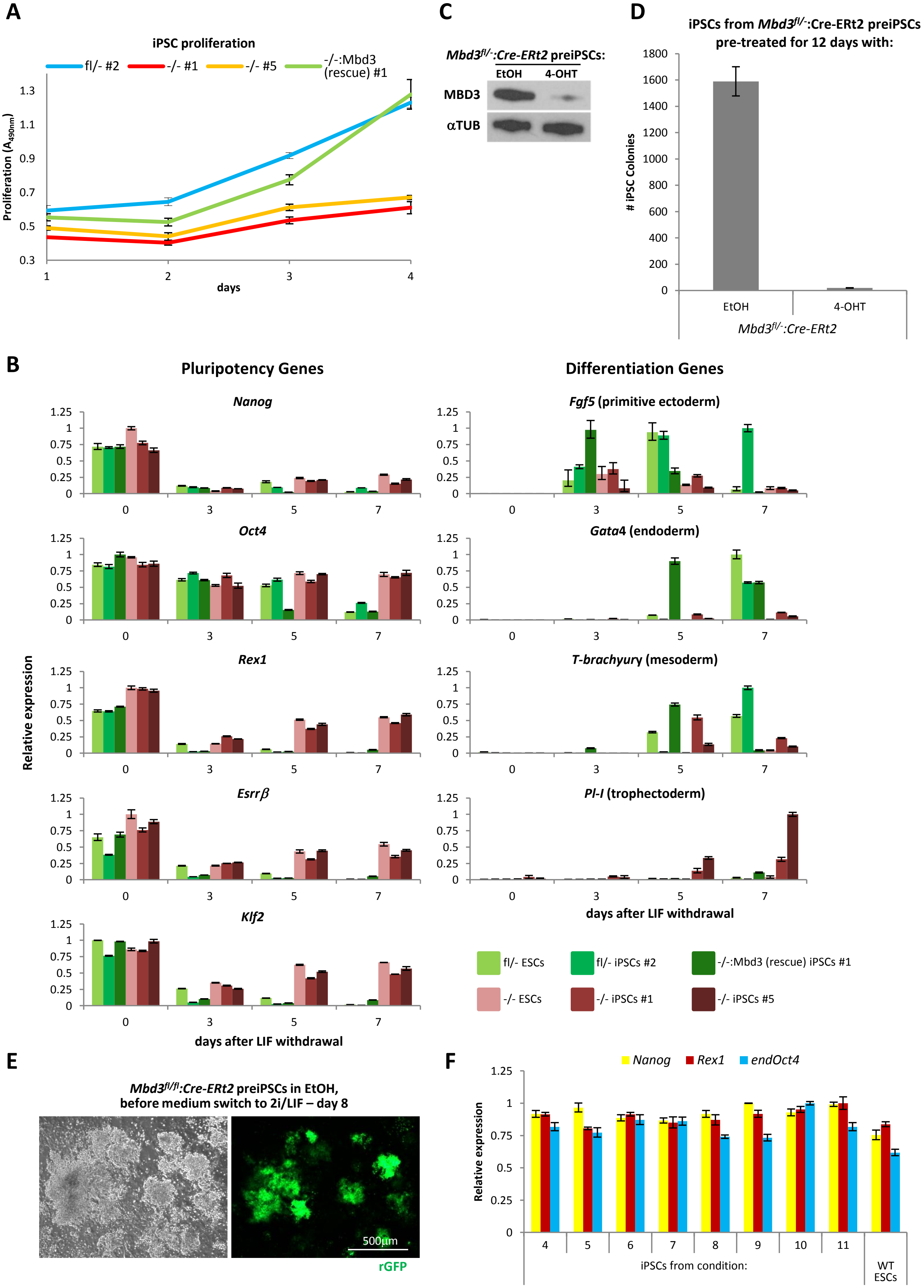
A
iPSC proliferation
fl/- #2
-/- #1
-/- #5
-/-:Mbd3 (rescue) #1
Proliferation (A490nm)
days
C
Mbd3fl/-:Cre-ERt2 preiPSCs:
EtOH
4-OHT
MBD3
αTUB
D
iPSCs from Mbd3fl/-:Cre-ERt2 preiPSCs pre-treated for 12 days with:
# iPSC Colonies
EtOH
4-OHT
Mbd3fl/-:Cre-ERt2
B
Pluripotency Genes
Differentiation Genes
Nanog
Oct4
Rex1
Esrrβ
Klf2
Relative expression
days after LIF withdrawal
Fgf5 (primitive ectoderm)
Gata4 (endoderm)
T-brachyury (mesoderm)
Pl-I (trophectoderm)
days after LIF withdrawal
fl/- ESCs
fl/- iPSCs #2
-/-:Mbd3 (rescue) iPSCs #1
-/- ESCs
-/- iPSCs #1
-/- iPSCs #5
E
Mbd3fl/fl:Cre-ERt2 preiPSCs in EtOH, before medium switch to 2i/LIF – day 8
500μm
rGFP
F
Nanog
Rex1
endOct4
Relative expression
iPSCs from condition:
WT ESCs

**Figure S2, related to Figure 2 - Mbd3 is required for efficient iPSC generation.**

**(A)** Cell proliferation analysis of *Mbd3*$^{fl/-}$, *Mbd3*$^{-/-}$ and *Mbd3*$^{-/-}$*:Mbd3* iPSCs culture in 2i/LIF conditions. **(B)** qRT-PCR analysis of pluripotency- and differentiation-associated genes during differentiation as embryoid bodies of *Mbd3*$^{fl/-}$, *Mbd3*$^{-/-}$, *Mbd3*$^{-/-}$*:Mbd3* iPSCs and ESCs controls. **(C)** Western blot analysis of MBD3 and $\alpha$-TUBULIN ($\alpha$TUB) protein levels in the *Mbd3*$^{fl/-}$*:Cre-ERt2* preiPSC treated with 4-OHT or EtOH for 12 days. **(D)** Quantification of iPSCs colonies generated from *Mbd3*$^{fl/-}$ preiPSCs stably transformed with pCAG-Cre-ERt2 and pre-treated with 4-OHT or EtOH for 12 days while cultured in S+LIF. Medium was switched from S+LIF to 2i/LIF 24h after plating. No 4-OHT or EtOH was added during 2i/LIF culture. Colony number is per 1.0 x $10^5$ preiPSCs plated. **(E)** Phase and GFP images of *Mbd3*$^{fl/fl}$*:Cre-ERt2* preiPSC colonies 8 days after transduction with rOKM + rGFP in presence of EtOH. Images taken before medium switch to 2i/LIF, day 8. **(F)** qRT-PCR analysis of pluripotency-associated genes in iPSCs generated from *Mbd3*$^{fl/fl}$*:Cre-ERt2* NSCs with *Mbd3* deletion at different time points of reprogramming, as described in Figure 2C. qRT-PCR values are normalized to *Gapdh* value and shown as relative to the highest value. The error bars indicate STDEV.

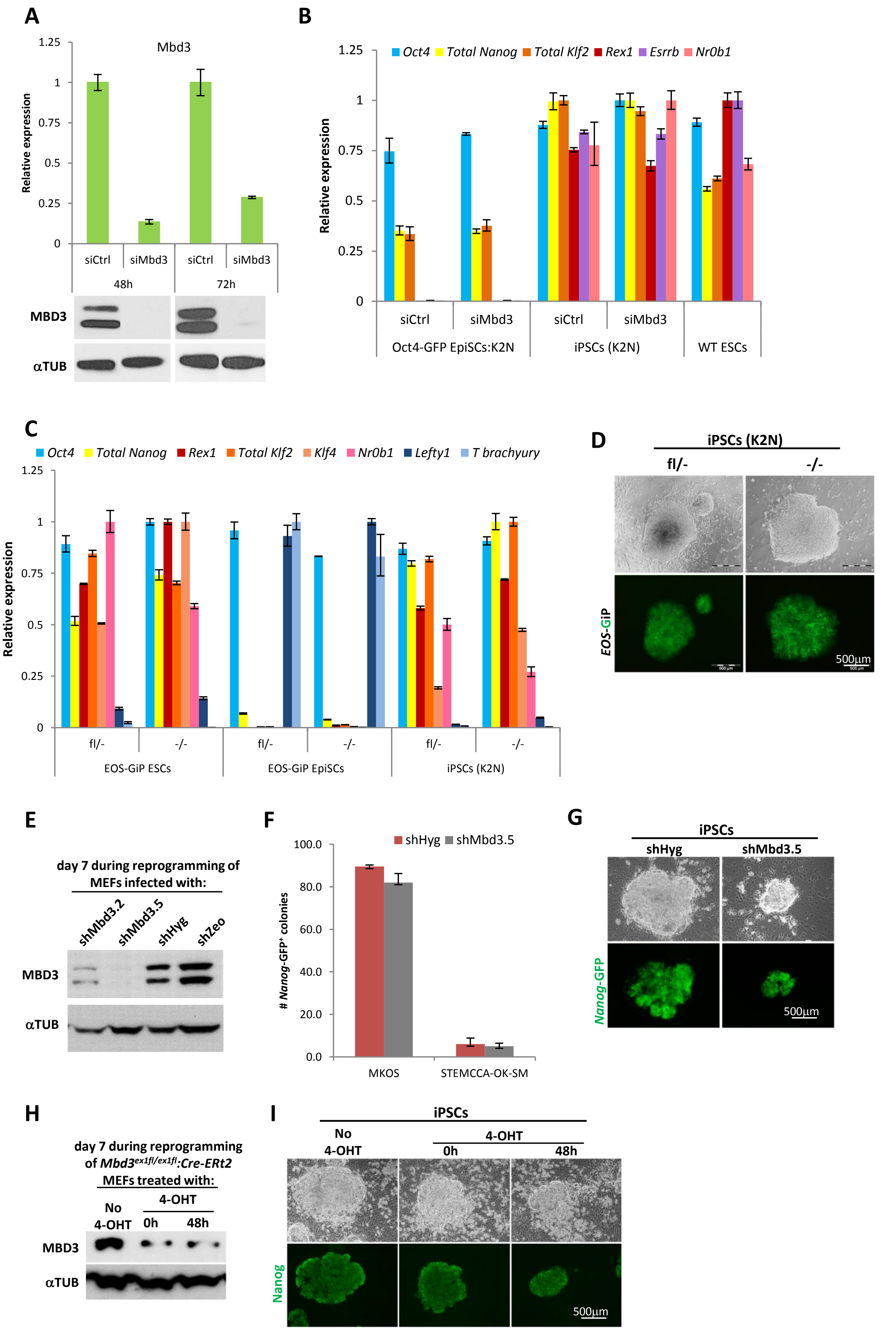
A
Mbd3
Relative expression
1.25
1
0.75
0.5
0.25
0
siCtrl
siMbd3
siCtrl
siMbd3
48h
72h
MBD3
αTUB
B
Oct4 Total Nanog Total Klf2 Rex1 Esrrb Nr0b1
Relative expression
1.25
1
0.75
0.5
0.25
0
siCtrl
siMbd3
siCtrl
siMbd3
Oct4-GFP EpiSCs:K2N
iPSCs (K2N)
WT ESCs
C
Oct4 Total Nanog Rex1 Total Klf2 Klf4 Nr0b1 Lefty1 T brachyury
Relative expression
1.25
1
0.75
0.5
0.25
0
fl/-
-/-
fl/-
-/-
fl/-
-/-
EOS-GiP ESCs
EOS-GiP EpiSCs
iPSCs (K2N)
D
iPSCs (K2N)
fl/-
-/-
EOS-GiP
500µm
E
day 7 during reprogramming of MEFs infected with:
shMbd3.2
shMbd3.5
shHyg
shZeo
MBD3
αTUB
F
shHyg shMbd3.5
# Nanog-GFP+ colonies
100.0
80.0
60.0
40.0
20.0
0.0
MKOS
STEMCCA-OK-SM
G
iPSCs
shHyg
shMbd3.5
Nanog-GFP
500µm
H
day 7 during reprogramming of Mbd3ex1fl/ex1fl:Cre-ERt2 MEFs treated with:
No 4-OHT
4-OHT
0h
48h
MBD3
αTUB
I
iPSCs
No 4-OHT
4-OHT
0h
48h
Nanog
500µm

**Figure S3, related to Figure 3 - Requirement of Mbd3 in other reprogramming systems**

**(A)** qRT-PCR and western blot analysis of *Mbd3* transcript and MBD3 and $\alpha$-TUBULIN ($\alpha$TUB) protein levels, respectively, 48h and 72h after siRNA-mediated KD. **(B)** qRT-PCR analysis of *Oct4*, total *Nanog*, *Rex1*, *Esrr$\beta$*, total *Klf2* and *Nr0b1* expression in Klf2-2A-Nanog transfected *Oct4*-GFP EpiSCs and derived from them iPSCs after siCtrl and siMbd3 transfection in 2i/LIF. **(C)** qRT-PCR analysis of *Oct4*, total *Nanog*, *Rex1*, total *Klf2*, *Klf4*, *Nr0b1*, *Lefty1* and *T (brachyury)* expression in parental *Mbd3*$^{fl/-}$ and *Mbd3*$^{-/-}$ *EOS*-GiP ESCs, *Mbd3*$^{fl/-}$ and *Mbd3*$^{-/-}$ *EOS*-GiP EpiSCs obtained from them, and EpiSC-derived iPSCs. qRT-PCR values are normalized to *Gapdh* value and shown as relative to the highest value. **(D)** Phase and *EOS*-GiP (GFPiresPuro under the control of early transposon promoter and Oct4 and Sox2 enhancers) images of *Mbd3*$^{fl/-}$ and *Mbd3*$^{-/-}$ iPSCs generated from EpiSCs. **(E)** Western blot analysis of MBD3 and $\alpha$-TUBULIN ($\alpha$TUB) protein levels at day 7 of reprogramming of MEFs infected with shMbd3 or control shRNA against Hygromycin or Zeocin resistant genes (shHyg or shZeo). Over 90% of MBD3 knockdown was observed. **(F)** Quantification of *Nanog*-GFP$^{+}$ iPSCs colonies generated by piggyBac MEF reprogramming using two different reprogramming cassettes, MKOS and STEMCCA-OK-SM. *Mbd3* KD using shRNA was carried out 24h after induction of the reprogramming cassettes. The error bars indicate STDEV. **(G)** Phase and *Nanog*-GFP images of iPSCs derived from MEFs transduced with lentiviruses encoding shHyg or shMbd3.5. **(H)** Western blot analysis of MBD3 and $\alpha$-TUBULIN ($\alpha$TUB) protein levels at day 7 during reprogramming of *Mbd3*$^{ex1fl/ex1fl:}$*Cre-ERt2* MEFs treated with 4-OHT 0h or 48h after induction of the reprogramming cassettes. About 80% of MBD3 knockdown was observed. **(I)** Phase and Nanog immunofluorescence images of the Nanog-positive iPSC colonies derived from *Mbd3*$^{ex1fl/ex1fl:}$*Cre-ERt2* treated with 4-OHT 0h or 48h after induction of the reprogramming cassettes.

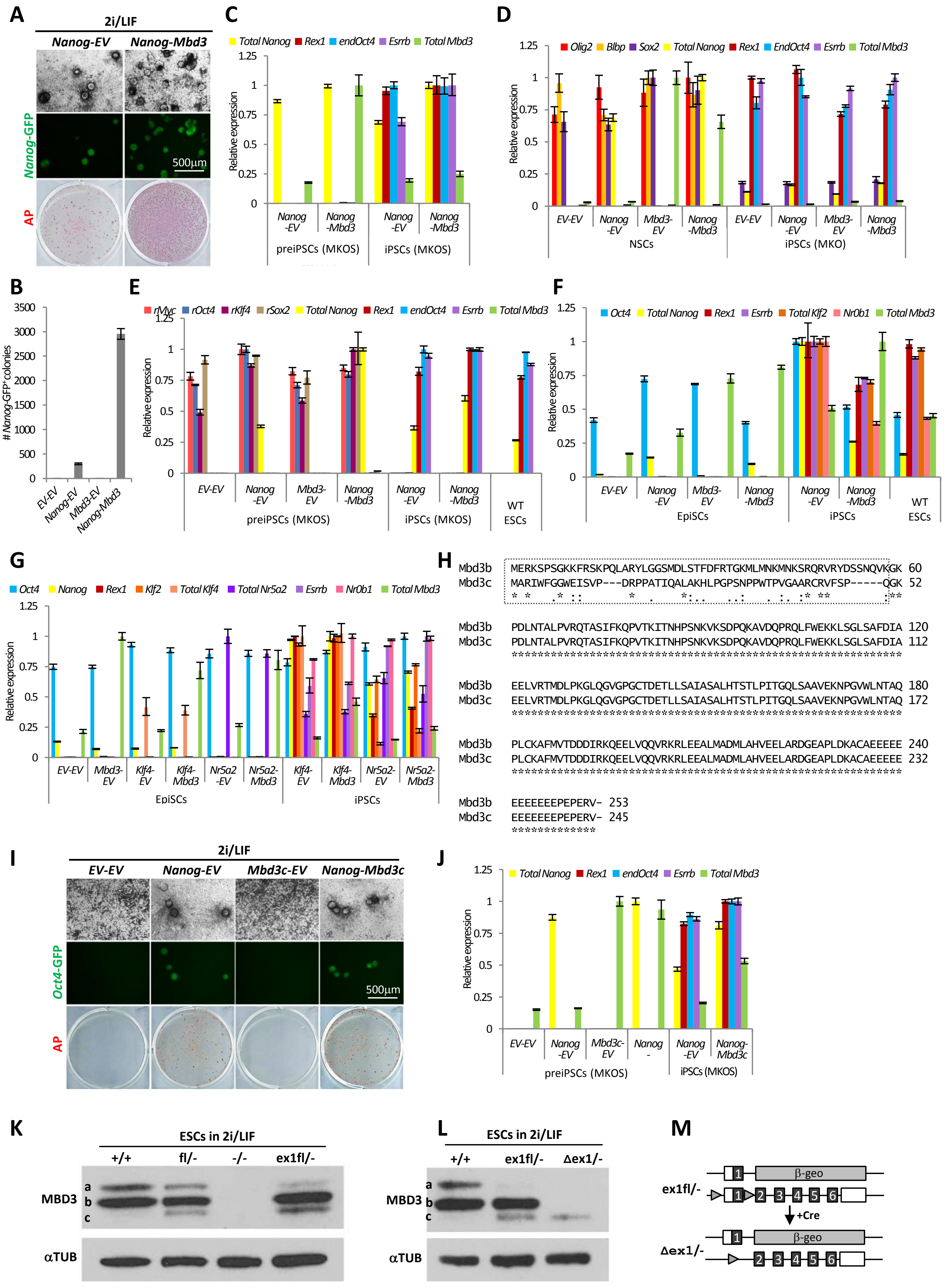
A
2i/LIF
Nanog-EV
Nanog-Mbd3
Nanog-GFP
500µm
AP
C
Total Nanog
Rex1
endOct4
Esrrb
Total Mbd3
Relative expression
preiPSCs (MKOS)
iPSCs (MKOS)
D
Olig2
Blbp
Sox2
Total Nanog
Rex1
EndOct4
Esrrb
Total Mbd3
EV-EV
Nanog -EV
Mbd3- EV
Nanog -Mbd3
NSCs
iPSCs (MKO)
B
# Nanog-GFP+ colonies
EV-EV
Nanog-EV
Mbd3-EV
Nanog-Mbd3
E
rMyc
rOct4
rKlf4
rSox2
Total Nanog
Rex1
endOct4
Esrrb
Total Mbd3
preiPSCs (MKOS)
iPSCs (MKOS)
WT ESCs
F
Oct4
Total Nanog
Rex1
Esrrb
Total Klf2
Nr0b1
Total Mbd3
EpiSCs
iPSCs
WT ESCs
G
Oct4
Nanog
Rex1
Klf2
Total Klf4
Total Nr5a2
Esrrb
Nr0b1
Total Mbd3
EV-EV
Mbd3- EV
Klf4- EV
Klf4- Mbd3
Nr5a2 -EV
Nr5a2- Mbd3
EpiSCs
iPSCs
H
Mbd3b MERKSPSGKKFRSKPQLARYLGGSMDLSTFDFRTGKMLMNKMNKSRQRVRYDSSNQVKGK 60
Mbd3c MARIWFGGWEISVP---DRPPATIQALAKHLPGPSNPPWTPVGAARCRVFSP-----QGK 52
Mbd3b PDLNTALPVRQTASIFKQPVTKITNHPSNKVKSDPQKAVDQPRQLFWEKKLSGLSAFDIA 120
Mbd3c PDLNTALPVRQTASIFKQPVTKITNHPSNKVKSDPQKAVDQPRQLFWEKKLSGLSAFDIA 112
Mbd3b EELVRTMDLPKGLQGVGPGCTDETLLSAIASALHTSTLPITGQLSAAVEKNPGVWLNTAQ 180
Mbd3c EELVRTMDLPKGLQGVGPGCTDETLLSAIASALHTSTLPITGQLSAAVEKNPGVWLNTAQ 172
Mbd3b PLCKAFMVTDDDIRKQEELVQQVRKRLEEALMADMLAHVEELARDGEAPLDKACAEEEEE 240
Mbd3c PLCKAFMVTDDDIRKQEELVQQVRKRLEEALMADMLAHVEELARDGEAPLDKACAEEEEE 232
Mbd3b EEEEEEEPEPERV- 253
Mbd3c EEEEEEEPEPERV- 245
I
2i/LIF
EV-EV
Nanog-EV
Mbd3c-EV
Nanog-Mbd3c
Oct4-GFP
500µm
AP
J
Total Nanog
Rex1
endOct4
Esrrb
Total Mbd3
Mbd3c- EV
Nanog -
Nanog- Mbd3c
preiPSCs (MKOS)
iPSCs (MKOS)
K
ESCs in 2i/LIF
+/+
fl/-
-/-
ex1fl/-
MBD3
a
b
c
αTUB
L
ESCs in 2i/LIF
+/+
ex1fl/-
Δex1/-
M
ex1fl/-
β-geo
+Cre
Δex1/-

**Figure S4, related to Figure 4 - Overexpression of Mbd3/NuRD facilitates Nanog-mediated reprogramming.**

**(A)** Phase and GFP images and AP staining of the iPSCs formed from preiPSCs overexpressing respective transgenes. It is to note that the expression level of Nanog transgene in the cell lines are similar (refer to Figure S4D). **(B)** Quantification of *Nanog*-GFP$^{+}$ colonies in Figure S4A, after 12 days of 2i/LIF culture. Colony numbers are per 1.0 x $10^{5}$ preiPSCs. **(C)** qRT-PCR analysis of pluripotency-associated genes and *Mbd3* in transgenic preiPSCs used in Figures S4A-B, and corresponding derived iPSCs. **(D)** qRT-PCR analysis of NSC- and pluripotency-associated genes, and *Mbd3* in transgenic NSCs and corresponding derived iPSCs. **(E)** qRT-PCR analysis of retroviral transgenes, pluripotency-associated, and *Mbd3* in preiPSCs used in Figures 4E-H, and corresponding derived iPSCs. **(F)** qRT-PCR analysis of pluripotency-associated genes and *Mbd3* in transgenic EpiSCs and corresponding derived iPSCs. **(G)** qRT-PCR analysis of pluripotency-associated genes and *Mbd3* in EpiSCs transfected with *Klf4* or *Nr5a2*, and corresponding derived iPSCs. **(H)** ClustalW2 sequence alignment between Mbd3b isoform (cDNA used in this study, unless stated otherwise) and Mbd3c isoform (http://www.ebi.ac.uk/Tools/msa/clustalw2/). The isoforms differ in their N-terminus sequence, because of different translation start sites, Mbd3c being 8 amino acids shorter (dotted box). **(I)** Phase and GFP images and AP staining of the iPSCs formed from preiPSCs overexpressing respective transgenes. **(J)** qRT-PCR analysis of pluripotency-associated genes and *Mbd3* in transgenic preiPSCs and corresponding derived iPSCs. qRT-PCR values are normalized to *Gapdh* value and shown as relative to the highest value. The error bars indicate STDEV. **(J-L)** Western blot analysis of MBD3 and α-TUBULIN (αTUB) protein levels in ESC lines with different *Mbd3* genotypes cultured in 2i/LIF conditions: (K) *Mbd3*$^{+/+}$, *Mbd3*$^{fl/-}$, *Mbd3*$^{-/-}$ (derived from *Mbd3*$^{fl/-}$ after Cre-mediated deletion), and *Mbd3*$^{ex1fl/-}$; (L) *Mbd3*$^{+/+}$,

*Mbd3*$^{ex1fl/-}$ and *Mbd3*$^{\Delta ex1/-}$ (derived from *Mbd3*$^{ex1fl/-}$ after Cre-mediated deletion). Mbd3 isoforms a-c are indicated. **(M)** Schematic representation of *Mbd3* loci in *Mbd3*$^{ex1fl/-}$ and *Mbd3*$^{\Delta ex1/-}$ ESCs. Exons are indicated as dark grey boxes, non-coding sequences as unfilled boxes and light grey triangles represent loxP sites. *Mbd3* exon 1 deletion creates a hypomorphic MBD3 protein, with lower molecular weight.

## Supplemental experimental procedures

**Plasmids:** pMX-Klf4, pMX-Oct4, pMX-c-Myc, pMX-Sox2, pMX-GFP and pLKO.1 (from Addgene repository); pPB-CAG-Nanog-pA-pgk-hph, pPB-CAG-DEST-pA-pgk-hph, pPB-CAG-Mbd3b-pA-pgk-bsd, pPB-CAG-Mbd3c-pA-pgk-bsd, pPB-CAG-Klf4-pA-pgk-hph, pPB-CAG-Nr5a2-pA-pgk-hph, pPB-CAG-DEST-pA-pgk-bsd, pPB-CAG-Klf2.2A.Nanog-Cherry-pA-pgk-zeo, pPB-CAG-Nr5a2-pA-pgk-hph, pCAG-Cre-ires-Puro and pCAG-Mbd3bihygro (made in the lab); pCAG-Cre-ERt2$^{NLS}$-IRES-BSD (from Joerg Betschinger); pPB-EOS-GFP-ires-Puro (pPB-EOS-GiP) (from Ge Guo); pCyL43 (PBase) and pPB-CAG-rtTA (from Sanger Institute's plasmid repository); PB-TAP IRI attP2LMKOSimO (O'Malley et al., 2013), PB-TAP IRI tetO-STEMCCAimO (Oct4-F2A-Klf4-ires-Sox2-E2A-c-Myc) and PB-TAP MKOSimO were generated by transferring STEMCCA reprogramming cassette into PB-TAP IRI piggyBac backbone with ires-mOrange (O'Malley et al., 2013; Sommer et al., 2009), and MKOSimO cassette into PB-tetO backbone (Woltjen et al., 2009), respectively. Sequences of the plasmids are available upon request.

**Generation of transgenic cell lines:** NSCs and preiPSCs were transfected using Amaxa Nucleofection Technology (Lonza AG). 2 x $10^6$ cells were used per transfection. ESCs, iPSCs and EpiSCs were transfected in suspension using Lipofectamine 2000 (Invitrogen). Both protocols were performed according to manufacturer's instructions. PiggyBac transposon (pPB) plasmids were co-transfected with piggyBac transposase expression vector pBase (mixture of 1:1) to generate stable cell lines. Selection for transgenes was applied for at least three passages before setting up experiments.

**siRNA knock-down:** *Mbd3* knock-down was carried out using Flexitube siRNAs (Qiagen) against *Mbd3* (Table below). Four different siRNAs (Mm_Mbd3_1, 2, 3 and 5) were

individually tested and three of them were chosen to be used as a pool in subsequent experiments (Mm_Mbd3_1 - SI00206836, Mm_Mbd3_3 - SI00206850 and Mm_Mbd3_5 - SI02740045). AllStars Negative Control siRNA (1027280) was used as a control. The final concentration of siRNAs for transfection was 0.2 μM/cm$^2$. For reprogramming experiments, EpiSCs were transfected with the siRNAs using the Lipofectamine RNAiMAX reagent (Life Technologies) according to manufacturer's protocol. The media was switched to 2i/LIF 24h after transfection.

**NSC and MEF reprogramming:** To generate retroviruses encoding reprogramming factors, 2 x 10$^6$ PLAT-E cells (per transfection) were seeded in 10 cm dishes and transfected the next day with 9 μg of pMXs plasmids (OKM or OKMS in case of NSCs and MEFs, respectively; where indicated, pMXs-GFP was also used) using FuGENE 6 transfection reagent (Roche) according to manufacturer's instructions. The medium was switched to S+LIF the next day. The retrovirus-containing supernatants from PLAT-E cultures were collected 48 hours post-transfection and filtered using 0.45 μm filters. Polybrene was added to the filtered supernatants to a final concentration of 4 μg/ml. The mixture was then applied to the plated NSCs or MEFs. In case of NSCs, 24h after incubation, the virus-containing medium was replaced with Egf+Fgf2 medium for 2-3 days, after which the cells were switched to S+LIF medium to enable preiPSCs (reprogramming intermediates) formation. MEFs were maintained in S+LIF throughout. The emergent preiPSCs were then switched to 2i/LIF medium to induce complete reprogramming. Where indicated in the text, preiPSCs were passaged and transfected at the preiPSC stage, plated in S+LIF and switched to in 2i/LIF conditions 1-2 days later. *Nanog*-GFP MEF derived preiPSCs were chosen to address synergy between *Nanog* and *Mbd3* during reprogramming, since they convert to naïve pluripotency very inefficiently in 2i/LIF conditions, unless transfected with additional

factors (cells used in (Costa et al., 2013)). Where cells contained a reprogramming reporter (*Nanog*-GFPiresPuro or *Oct4*-GFPiresPuro), puromycin was added to 2i/LIF cultures six days after medium switch. Reprogramming experiments were ended 12 days after medium switch to 2i/LIF.

**EpiSC reprogramming:** Transgenic EpiSCs were plated in Fgf2/Act.A medium and switched to 2i/LIF conditions the next day. Once medium is switched to 2i/LIF EpiSCs no longer proliferate, unless they undergo reprogramming, making resulting iPSC colonies representative of initial plated EpiSC numbers. Where cells contained a reprogramming reporter (*Oct4*-GFPiresPuro of *EOS*-GFPiresPuro), 1 μg/ml puromycin was added to 2i/LIF cultures six days after medium switch. Reprogramming experiments were ended 12 days after medium switch to 2i/LIF. Number of EpiSCs plated differ from experiment to experiment and are indicated in the figure legends.

**piggyBac transposon reprogramming**: The PB-TAP IRI attP2LMKOSimO or PB-TAP IRI tetO-STEMCCAimO (500 ng), pPB-CAG-rtTA (500 ng) and pCyL43 piggyBac transposase expression vector (500 ng) were introduced into the MEFs with *Nanog-GFP* reporter (Chambers et al., 2007) seeded at 1 x $10^5$ cells/wells in 6-well plates on the day before transfection using 6 μl of FugeneHD (Promega). Twenty-four hours later, culture medium was changed to S+LIF medium supplemented with 1.0 μg/ml doxycycline (dox), vitamin C (Sigma) (10 μg/ml) and Alk inhibitor A 83-01 (TOCRIS Bioscience) (500 nM) (+DVA). This medium was changed every two days until day 13 of reprogramming. Lentiviral infection using pLKO.1 (Addgene) with shRNA expression against *Mbd3* (shMbd3.2 and shMbd3.5), Hygromycin and Zeocin resistant genes (shHyg and shZeo) was carried out 24 hours after dox administration. Cell lysates from one of the triplicate at day 7 of reprogramming were used for Western blotting analysis to confirm MBD3 knockdown. For *Mbd3* exon1 deletion,

retroviral Cre-ERt2 expression vector was infected 24 hours after PB-TAP MKOSimO piggybac transfection with Fugene. At the same time culture medium was changed to S+LIF +DVA medium. 4-OHT was added at this point or 48 hours later, and kept in the culture medium for 48 hours. Cell lysates from one of the triplicate at day 7 of reprogramming were used for Western blotting analysis to confirm MBD3 depletion.

**Embryoid Body differentiation:** 1.5 x $10^6$ cells were plated in non-adherent 10 cm bacterial dishes in serum minus LIF medium. Samples were collected at day 3, 5 and 7 of differentiation and analysed by qRT-PCR.

**Blastocyst injection, chimera generation and germline transmission assessment:** For blastocyst injection standard microinjection methodology using host blastocysts of C57BL/6 strain was employed. Floxed pPB transgenes were excised using TAT-Cre treatment before injection. Injected blastocysts were transferred to recipient mice to assess the contribution to chimeras. Generated chimeras were back-crossed with C57BL/6 mice to assess germline transmission.

**Alkaline Phosphatase staining:** Cells were fixed with a citrate-acetone-formaldehyde solution and stained for 30min using the Alkaline Phosphatase kit (Sigma-Aldrich) according to manufacturer's instructions.

**Flow cytometry and imaging:** Flow cytometry analysis was performed using an LSRFortessa analyser (BD). Cell sorting was performed using a MoFlo high-speed cell sorter. GFP was excited by a 488nm laser and detected using a 530/30 filter. All data analysis was performed using FlowJo software. Live cells were imaged with inverted Olympus IX51 microscope supplied with the Leica DFC310 FX digital colour camera, and processed with Leica software.

**Western blotting:** Protein extracts were obtained by incubation of cells in RIPA buffer (PBS, 1% NP40, 0.1% SDS, 0.1 mM EDTA supplemented with proteinase inhibitors, Roche). Total protein was quantified using BCA protein assay kit (Thermo Scientific) and the same amount of protein was loaded into gels (NuPAGE 10% Bis-Tris, Novex Life Technologies) for all the samples. Protein transfer was carried out using the iBlot system (iBlot Gel transfer stack nitrocellulose, Novex Life Technologies). Primary antibodies used: rabbit polyclonal MBD3 antibody (Bethyl, A302-528A, 1:2000), goat polyclonal MTA2 antibody (C-20) (Santa Cruz, sc-9447, 1:1000) and mouse monoclonal antibody α-TUBULIN (Abcam, ab7291, 1:5000). Secondary HRP-conjugated antibodies used: anti-rabbit (GE, NA934VS, 1:10000), anti-mouse (GE, NA931VS) and anti-goat (Santa Cruz, sc-2020, 1:2000). Blocking was carried out in 5% milk/0.1% tween in PBS (blocking solution) for 1h at RT. Primary antibodies were diluted in blocking solution and incubated with the membrane overnight at $4^0$C. Three washing steps of 10 min were carried out with blocking solution after primary antibody incubation. Secondary antibodies were diluted in blocking solution and incubated with membranes for 1h at RT. Then three washing steps of 20min were performed with 0.1% tween in PBS. Membranes were developed using the ECL Prime detection kit (GE Healthcare) according to manufacturer's instructions. Membrane re-probing with another primary antibody was carried out after stripping the membrane with 100 mM β-Mercaptoethanol, 2% SDS and 62.5 mM Tris-HCl solution in water.

**RNA isolation, cDNA synthesis and qRT-PCR:** Total RNA was isolated from cells using the RNeasy mini kit (QIAGEN) in accordance with manufacturer's protocol. After purification, 1 μg of total RNA was reverse transcribed into cDNA using the SuperScript III First-Strand Synthesis SuperMix kit (Life Technologies). 10 ng of cDNA was used for qRT-PCR reactions that were set up in triplicates using either TaqMan Universal PCR Master Mix or

Fast SYBR Green Master Mix (Life Technologies). TaqMan gene expression assays (Applied Biosystems) or specific primers (Table below) were used for each gene analysed. qRT-PCR experiments were performed using StepOnePlus Real Time PCR System (Applied Biosystems). Delta Ct values with *Gapdh* were calculated and brought to power -2. Where indicated, the values were normalized to the highest value. Error bars represent ±S.D. of technical triplicates.

**Cell proliferation analysis:** Cell proliferation was assessed by counting cells every 24h using the Vi-Cell XR Cell Viability analyser (Beckman Coulter). For iPSCs proliferation analysis, 2000 cells were plated per 96-well plate and assayed with CellTiter 96 AQueous One Solution Cell Proliferation Assay (Promega) every 24h.

**Primers and shRNA oligos used in the study.**

| | |
|---|---|
| ***Mbd3* genotyping primers** | |
| Mbd3 gen. FP | ACTGCTCCAGCTTGGTACAG |
| Mbd3 gen. RP | AATCAGATCACTTCAGCTCC |
| **Primers used with SYBR green** | |
| Mbd3 FP | AGAAGAACCCTGGTGTGTGG |
| Mbd3 RV | TGTACCAGCTCCTCCTGCTT |
| Pl-1 FP | ATTTTGACTACCCTGCTTGGTCT |
| Pl-1 RP | TCTACATAACTGAGGAGGGGAAAG |
| GAPDH FP | CCCACTAACATCAAATGGGG |
| GAPDH RP | CCTTCCACAATGCCAAAGTT |
| Olig2 FP | CTGCTGGCGCGAAACTACAT |
| Olig2 RP | CGCTCACCAGTCGCTTCAT |
| Blbp FP | AGACCCGAGTTCCTCCAGTT |
| Blbp RP | ATCACCACTTTGCCACCTTC |
| Sox2 FP | TCCAAAAACTAATCACAACAATCG |
| Sox2 RP | GAAGTGCAATTGGGATGAAAA |
| Nr5a2 FP | CCAGAAAACATGCAAGTGTCTCAA |
| Nr5a2 RP | CGTGAGGAGACCGTAATGGTA |

| | |
|---|---|
| **Applied Biosystems Taqman probes** | |
| Nanog | Mm02384862_g1 |
| Rex1 | Mm03053975_g1 |
| Klf4 | Mm00516104_m1 |
| Klf2 | Mm01244979_g1 |
| Fgf5 | Mm00438919_m1 |
| Lefty1 | Mm00438615_m1 |
| T-Brachyury | Mm01318252_m1 |
| GAPDH | 4352339E |
| Esrrβ | Mm00442411_m1 |
| Gata4 | Mm00484689_m1 |
| Nr0b1 | Mm00431729_m1 |
| **Applied Biosystems custom Taqman probes** | |
| Retroviral Klf4 FP | TGGTACGGGAAATCACAAGTTTGTA |
| Retroviral Klf4 RP | GAGCAGAGCGTCGCTGA |
| Retroviral Klf4 probe | FAM-CCCCTTCACCATGGCTG-MGB |
| Retroviral Oct4 FP | TGGTACGGGAAATCACAAGTTTGTA |
| Retroviral Oct4 RP | GGTGAGAAGGCGAAGTCTGAAG |
| Retroviral Oct4 probe | FAM-CACCTTCCCCATGGCTG-MGB |
| Retroviral cMyc FP | TGGTACGGGAAATCACAAGTTTGTA |
| Retroviral cMyc RP | GGTCATAGTTCCTGTTGGTGAAGTT |
| Retroviral cMyc probe | FAM-CCCTTCACCATGCCCC-MGB |
| Retroviral Sox2 FP | TGGTACGGGAAATCACAAGTTTGTA |
| Retroviral Sox2 RP | GCCCGGCGGCTTCA |
| Retroviral Sox2 probe | FAM-CTCCGTCTCCATCATGTTAT-MGB |
| Endogenous Oct4 FP | TTCCACCAGGCCCCC |
| Endogenous Oct4 RP | GGTGAGAAGGCGAAGTCTGAAG |
| Endogenous Oct4 probe | FAM-CCCACCTTCCCCATGGCT-MGB |
| **siRNAs from Qiagen** | |
| Mm_Mbd3_1 - SI00206836 | CGGAAAGATGTTGATGAACAA |
| Mm_Mbd3_2 - SI00206843 | ACCGGTGACCAAGATCACCAA |

| | | |
|---|---|---|
| Mm_Mbd3_3 - SI00206850 | CAGGACCATGGACTTGCCCAA | |
| Mm_Mbd3_5 - SI02740045 | AAGTCACTTTCCTTCAATAAA | |
| AllStars Negative Control siRNA | Cat. No.: 1027280 | |
| **Upper strand oligos used for lentiviral knockdown vectors** | | |
| shMbd3.2 | Upper strand | CCGGGCGCTATGATTCTTCCAACCACTCGAGTGGTTGGAAGAATCATAGCGCTTTTT |
| | Bottom strand | AATTAAAAAGCGCTATGATTCTTCCAACCACTCGAGTGGTTGGAAGAATCATAGCGC |
| shMbd3.5 | Upper strand | CCGGAAGTCACTTTCCTTCAATAAACTCGAGTTTATTGAAGGAAAGTGACTTTTTTT |
| | Bottom strand | AATTAAAAAAAGTCACTTTCCTTCAATAAACTCGAGTTTATTGAAGGAAAGTGACTT |
| shHyg | Upper strand | CCGGGCGAAGAATCTCGTGCTTTCACTCGAGTGAAAGCACGAGATTCTTCGCTTTTT |
| | Bottom strand | AATTAAAAAGCGAAGAATCTCGTGCTTTCACTCGAGTGAAAGCACGAGATTCTTCGC |
| shZeo | Upper strand | CCGGGCCAAGTTGACCAGTGCCGTTCTCGAGAACGGCACTGGTCAACTTGGCTTTTT |
| | Bottom strand | AATTAAAAAGCCAAGTTGACCAGTGCCGTTCTCGAGAACGGCACTGGTCAACTTGGC |

## Supplemental References


Chambers, I., Silva, J., Colby, D., Nichols, J., Nijmeijer, B., Robertson, M., Vrana, J., Jones, K., Grotewold, L., and Smith, A. (2007). Nanog safeguards pluripotency and mediates germline development. Nature 450, 1230-U1238.

Costa, Y., Ding, J., Theunissen, T.W., Faiola, F., Hore, T.A., Shliaha, P.V., Fidalgo, M., Saunders, A., Lawrence, M., Dietmann, S., et al. (2013). NANOG-dependent function of TET1 and TET2 in establishment of pluripotency. Nature 495, 370-374.

O'Malley, J., Skylaki, S., Iwabuchi, K.A., Chantzoura, E., Ruetz, T., Johnsson, A., Tomlinson, S.R., Linnarsson, S., and Kaji, K. (2013). High-resolution analysis with novel cell-surface markers identifies routes to iPS cells. Nature 499, 88-91.

Sommer, C.A., Stadtfeld, M., Murphy, G.J., Hochedlinger, K., Kotton, D.N., and Mostoslavsky, G. (2009). Induced pluripotent stem cell generation using a single lentiviral stem cell cassette. Stem Cells 27, 543-549.

Woltjen, K., Michael, I.P., Mohseni, P., Desai, R., Mileikovsky, M., Hamalainen, R., Cowling, R., Wang, W., Liu, P.T., Gertsenstein, M., et al. (2009). piggyBac transposition reprograms fibroblasts to induced pluripotent stem cells. Nature 458, 766-U106.
