# Supplementary material for: MBD3/NuRD Facilitates Induction of Pluripotency in a Context-Dependent Manner
Source: Cell Stem Cell. 2014 Jul 3;15(1):102–10. doi: 10.1016/j.stem.2014.04.019 (PMC4082719; doi:10.1016/j.stem.2014.04.019)
Supplement: Document S2. Article plus Supplemental Information [file mmc2.pdf]

Cell Stem Cell
Short Article

MBD3/NuRD Facilitates Induction of Pluripotency in a Context-Dependent Manner

Rodrigo L. dos Santos,[1,2] Luca Tosti,[3] Aliaksandra Radzisheuskaya,[1] Isabel M. Caballero,[3,5] Keisuke Kaji,[3,4] Brian Hendrich,[1,4] and José C.R. Silva[1,4,*]
[1]Wellcome Trust – Medical Research Council Cambridge Stem Cell Institute and Department of Biochemistry, University of Cambridge, Tennis Court Road, Cambridge CB2 1QR, UK
[2]Doctoral Programme in Experimental Biology and Biomedicine, Centre for Neuroscience and Cell Biology and Institute for Interdisciplinary Research, University of Coimbra, 3030-789 Coimbra, Portugal
[3]MRC Centre for Regenerative Medicine, University of Edinburgh, Edinburgh BioQuarter, 5 Little France Drive, Edinburgh EH16 4UU, UK
[4]Co-senior author
[5]Present address: Laboratory of Molecular Neurobiology, Department of Medical Biochemistry and Biophysics, Karolinska Institute, Stockholm, 171 77 Sweden
*Correspondence: jcs64@cscr.cam.ac.uk
http://dx.doi.org/10.1016/j.stem.2014.04.019
This is an open access article under the CC BY license (http://creativecommons.org/licenses/by/3.0/).

SUMMARY

The Nucleosome Remodeling and Deacetylase (NuRD) complex is essential for embryonic development and pluripotent stem cell differentiation. In this study, we investigated whether NuRD is also involved in the reverse biological process of induction of pluripotency in neural stem cells. By knocking out MBD3, an essential scaffold subunit of the NuRD complex, at different time points in reprogramming, we found that efficient formation of reprogramming intermediates and induced pluripotent stem cells from neural stem cells requires NuRD activity. We also show that reprogramming of epiblast-derived stem cells to naive pluripotency requires NuRD complex function and that increased MBD3/NuRD levels can enhance reprogramming efficiency when coexpressed with the reprogramming factor NANOG. Our results therefore show that the MBD3/NuRD complex plays a key role in reprogramming in certain contexts and that a chromatin complex required for cell differentiation can also promote reversion back to a naive pluripotent cell state.

INTRODUCTION

Reprogramming of somatic cells to naive pluripotency can be robustly driven by the combined action of transcription factors and culture cues. Among the reprogramming transcription factors, OCT4 plays a central role, as it is sufficient and essential for the induction of pluripotent cells (Kim et al., 2009; Radzisheuskaya et al., 2013; Radzisheuskaya and Silva, 2014). OCT4 interactome studies in embryonic stem cells (ESCs) revealed members of the Nucleosome Remodeling and Deacetylase (NuRD) complex as its highest confidence interactors (Ding et al., 2012; Liang et al., 2008; Pardo et al., 2010; van den Berg et al., 2010). NuRD is composed of six core subunits with at least two enzymatic activities involved in gene regulation: histone deacetylase activity of HDAC1/2 subunits and ATP-dependent chromatin remodeling activity of Mi-2a/β subunits (Lai and Wade, 2011; McDonel et al., 2009). Methyl-CpG binding domain protein 3 (MBD3) is an essential scaffold protein of the NuRD complex, in the absence of which the complex is not assembled (Kaji et al., 2006; Zhang et al., 1999). Embryos lacking MBD3 die shortly after implantation (Hendrich et al., 2001; Kaji et al., 2007) and *Mbd3*-null ESCs are viable but show severely impaired lineage commitment and exhibit limited differentiation capacity (Kaji et al., 2006; Reynolds et al., 2012a, 2012b). Chromatin remodeling plays an important role in reprogramming to naive pluripotency (Apostolou and Hochedlinger, 2013; Papp and Plath, 2013). Because the NuRD complex is a high confidence interactor of Oct4 and a key regulator of developmental cell state transitions, we have investigated its involvement in the induction of pluripotency.

RESULTS

MBD3 Facilitates the Initiation of Reprogramming from Neural Stem Cells

To address the requirement of the NuRD complex in the reprogramming process, we established an $Mbd3^{-/-}$ clonal neural stem cell (NSC) line from $Mbd3^{fl/-}$ NSCs and an $Mbd3^{-/-}$ rescue NSC line ($Mbd3^{-/-}$:*Mbd3*) by stable transfection of an *Mbd3* transgene (Figures S1A–S1C available online). These NSC lines were transduced with retroviruses encoding *cMyc*, *Klf4*, and *Oct4* (rMKO) to initiate their reprogramming and were then switched to serum plus LIF (S+LIF) conditions (Figure 1A), which typically results in the formation of highly proliferative reprogramming intermediates, or preiPSCs (Silva et al., 2008). When we used retroviruses encoding *GFP* (rGFP), equal percentages of GFP^{+} cells were observed 72 hr after transduction of $Mbd3^{fl/-}$ or $Mbd3^{-/-}$ NSCs, indicating that *Mbd3* deletion does not affect transduction efficiency (Figures S1D and S1E). Strikingly, the kinetics of preiPSC emergence was markedly delayed in the $Mbd3^{-/-}$ cells. While *Mbd3*-expressing preiPSCs dominated the culture by day 4 posttransduction (d.p.t.), $Mbd3^{-/-}$

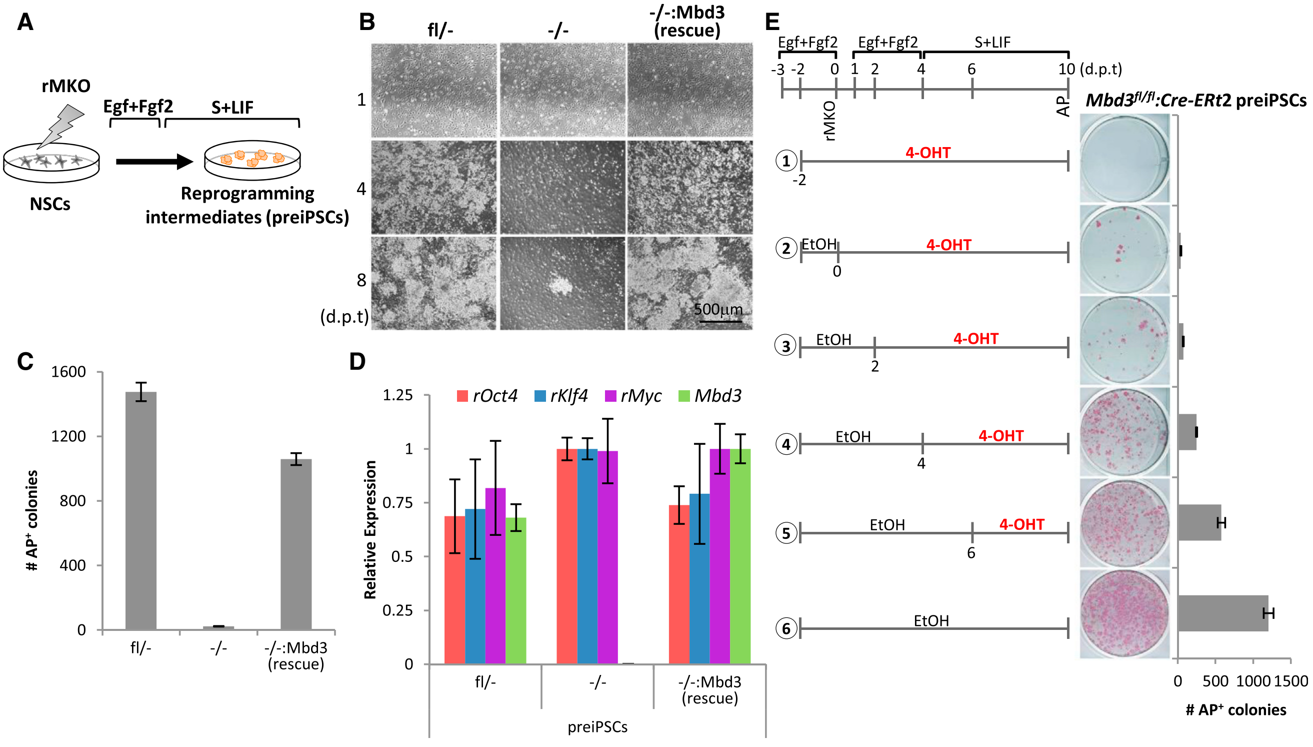

Figure 1. MBD3 Facilitates the Initiation of Reprogramming

(A) Experimental design used to address the kinetics and efficiency of initiation of reprogramming in NSCs with different *Mbd3* genotypes. NSCs were transduced with retroviruses encoding *cMyc*, *Klf4*, and *Oct4* (rMKO), maintained in Egf+Fgf2 medium for 3 days, and then switched to S+LIF medium.

(B) Phase images of the reprogramming intermediates (preiPSCs) emerging from $Mbd3^{fl/-}$, $Mbd3^{-/-}$, and $Mbd3^{-/-}$:*Mbd3* (rescue) NSCs at different days posttransduction (d.p.t.).

(C) Efficiency of preiPSC colony formation per 2.5×10^5 NSCs as assessed by alkaline phosphatase (AP) staining at day 9 posttransduction.

(D) qRT-PCR analysis of retroviral transgenes (*rOct4*, *rKlf4*, and *rMyc*) and *Mbd3* expression in the obtained preiPSCs maintained in S+LIF. Three independent NSCs transductions were carried out and gene expression was assessed 12 days after transduction. Values are normalized to *Gapdh* value and shown as relative to the highest value.

(E) Time course of MBD3 requirement during preiPSC formation. $Mbd3^{fl/fl}$ NSCs were stably transfected with pCAG-CreERt2 transgene, transduced with retroviral transgenes, and treated with 4-OHT at indicated time points to induce Cre-mediated deletion of the floxed alleles during reprogramming. Ethanol (EtOH) was used as a control. The encircled numbers correspond to different conditions. PreiPSC colony formation was assessed by AP staining at day 10 post-transduction and is presented as the number of colonies per 7.5×10^4 NSCs. The error bars indicate STDEV.

preiPSCs emerged only by 7–8 d.p.t. (Figure 1B). In addition, the number of emerging alkaline-phosphatase-positive (AP$^+$) $Mbd3^{-/-}$ preiPSC colonies was significantly reduced compared to parental and rescue cell lines (Figure 1C and Figure S1F). Nevertheless, it was possible to establish and expand $Mbd3^{-/-}$ preiPSCs, although less efficiently and with delayed kinetics. Both $Mbd3^{-/-}$ NSCs and *Mbd3*-null preiPSCs derived from them exhibited slower proliferation, consistent with previous reports of $Mbd3^{-/-}$ ESCs (Kaji et al., 2006; Sims and Wade, 2011) (Figures S1G and S1H). $Mbd3^{-/-}$ preiPSCs expressed slightly higher levels of retroviral transgenes compared to control cells (Figure 1D), suggesting that dosage of reprogramming factors is not the reason for the reduced efficiency of reprogramming initiation that we observed.

To further dissect the requirement for MBD3 in the initiation of reprogramming, we analyzed the effect of *Mbd3* deletion at different experimental time points. For this experiment, we stably transfected $Mbd3^{fl/fl}$ NSCs with *Cre-ERt2*, which enabled Cre-mediated excision of the floxed *Mbd3* alleles upon addition of 4-hydroxytamoxifen (4-OHT) (Figures S1I–S1L). We found that earlier removal of *Mbd3* reduced the number of preiPSC colonies formed (Figure 1E). We also obtained similar results after conditional deletion of *Mbd3* exon 1 (ex1fl) which removes all but a small amount of a truncated MBD3 protein isoform (MBD3C) (Aguilera et al., 2011; Kaji et al., 2006) (Figures S1M and S1N).

Taken together, these results demonstrate that lack of a functional NuRD complex strongly impairs the initiation of reprogramming from NSCs.

MBD3 Is Required for Efficient iPSC Generation from NSCs, preiPSCs, and EpiSCs

We then evaluated the role of MBD3 in later stages of reprogramming. To induce completion of the reprogramming process, $Mbd3^{fl/-}$, $Mbd3^{-/-}$, and rescued $Mbd3^{-/-}$:*Mbd3* preiPSCs were switched to serum-free medium containing LIF and inhibitors of both mitogen-activated protein kinase and glycogen synthase kinase-3 signaling (2i/LIF) (Silva et al., 2008), and the resulting iPSC colonies were scored 12 days later. We observed that the efficiency of conversion to naive pluripotency of $Mbd3^{-/-}$ preiPSCs is strongly reduced compared to

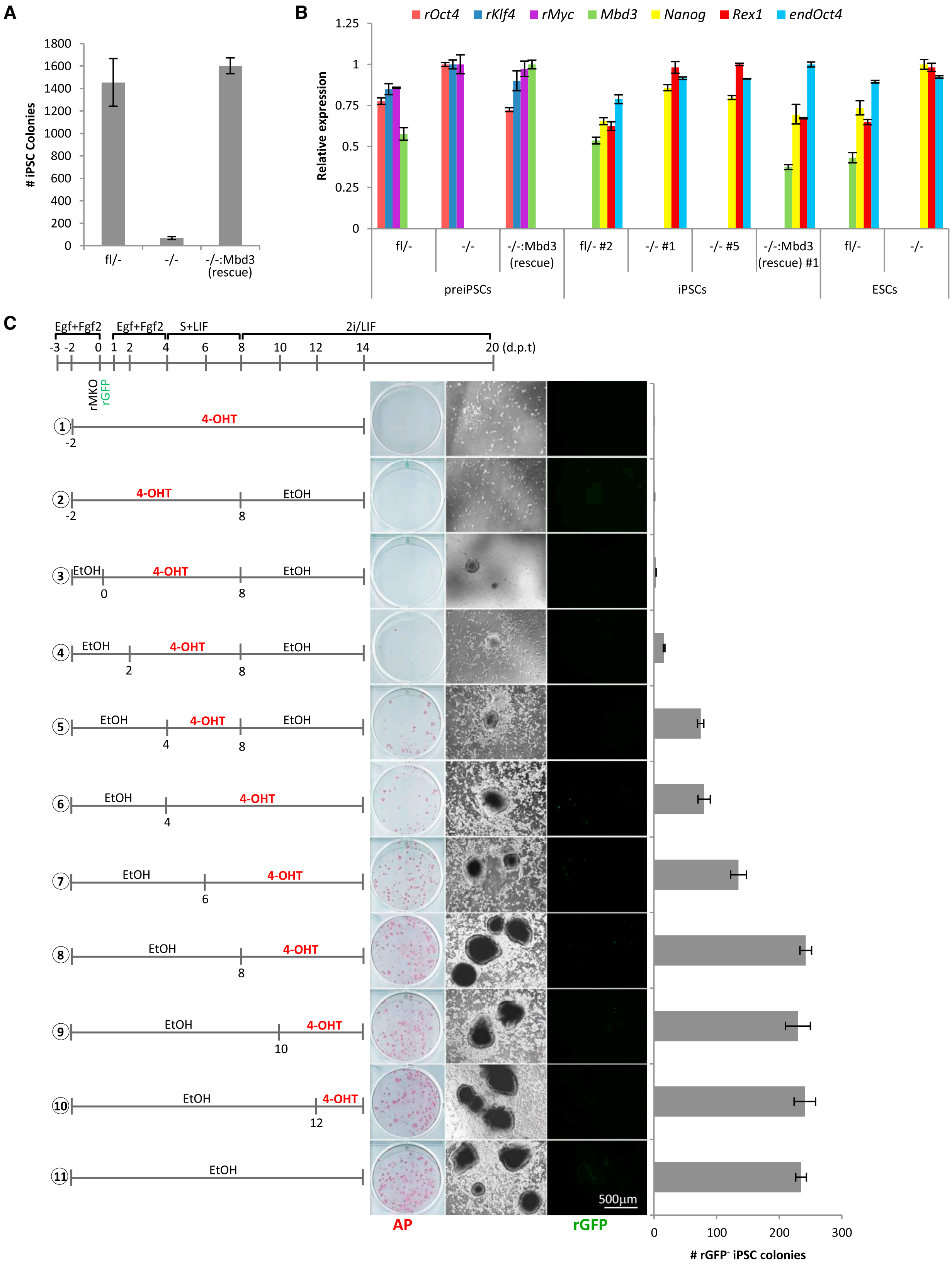

(legend on next page)

Mbd3$^{fl/-}$ and *Mbd3*$^{-/-}$*:Mbd3* preiPSCs (Figure 2A). The *Mbd3*$^{fl/-}$, *Mbd3*$^{-/-}$, and *Mbd3*$^{-/-}$*:Mbd3* iPSCs that were obtained could be expanded clonally in 2i/LIF, and they exhibited reactivation of the pluripotency transcriptional program and silencing of the retroviral reprogramming transgenes as expected (Figure 2B). The *Mbd3*$^{-/-}$ iPSCs were phenotypically similar to previously reported *Mbd3*-null ESCs (Kaji et al., 2006), exhibiting impaired embryoid body differentiation and slower proliferation (Figures S2A and S2B). We also observed that *Mbd3* deletion in an established preiPSC line before the 2i/LIF medium switch impaired reprogramming to naive pluripotency (Figures S2C and S2D).

Next we performed a time course experiment to define the time frame during reprogramming for which MBD3 is required. For this analysis, we transduced *Mbd3*$^{fl/fl}$*:Cre-ERt2* NSCs with rMKO and rGFP and treated them with 4-OHT at different experimental time points (Figure 2C). The growth medium was changed to S+LIF 4 days after transduction and subsequently, 4 days later, to 2i/LIF. The number of iPSC colonies exhibiting silencing of retroviral GFP expression was assessed 12 days after 2i/LIF medium switch (Figure 2C and Figure S2E). We observed that the number of iPSC colonies formed was proportional to the amount of time cells expressed MBD3 during the initiation phase of reprogramming (prior to 2i/LIF culture). We observed neither a reduction nor a gain of reprogramming efficiency when *Mbd3* was deleted at the 2i/LIF stage. Regardless of the stage of *Mbd3* deletion, the resulting iPSCs displayed a pluripotency-associated transcriptional signature (Figure S2F). Thus, our data suggest that MBD3 is specifically required for the initiation and intermediate stage of NSC reprogramming rather than establishment of pluripotency.

Epiblast stem cells (EpiSCs) can be reprogrammed to naive pluripotency by a combination of overexpression of at least one transcription factor, such as KLF4, KLF2, or NANOG, and the use of serum-free 2i/LIF medium, which not only promotes reprogramming of EpiSCs but also blocks their self-renewal (Guo et al., 2009; Silva et al., 2009). To examine the role of MBD3 in reprogramming in this context, we stably transfected wild-type EpiSCs carrying an *Oct4*-GFP reporter with piggyBac (PB) vectors constitutively expressing *Klf2* and *Nanog* (K2N) or *Klf4*, and we then transfected these with either small interfering RNA (siRNA) against *Mbd3* or control siRNA (Figure 3A). Strikingly, *Mbd3* knockdown led to a complete impairment of KLF4-mediated reprogramming and to a 6-fold reduction in the reprogramming ability of K2N (Figure 3B and Figures S3A and S3B). Similar results were obtained when EpiSCs with *Mbd3* genetic knockout were used (Figure 3C and Figures S3C and S3D).

All the results described above indicate that MBD3 is critical for efficient reprogramming in the contexts that we examined, contrasting with previous reports (Luo et al., 2013; Rais et al., 2013). To examine whether this difference is a reflection of the specific reprogramming systems that we used, we performed PB-mediated reprogramming of mouse embryonic fibroblasts (MEFs) combined with *Mbd3* depletion using two different approaches (Figure 3D). First, we used an *Mbd3* knockdown system in which *Nanog*-GFP MEFs were treated with doxycycline (DOX) for the induction of the MKOS or STEMCCA reprogramming cassettes (Kaji et al., 2009; Sommer et al., 2009) and cultured in S+LIF medium supplemented with vitamin C and Alki (Tgfβ signaling inhibitor). Twenty-four hours after induction they were transduced with lentiviruses expressing shRNA against *Mbd3* (Figure 3E and Figures S3E–S3G). Second, we depleted *Mbd3* by treating *Cre-ERt2*-transduced *Mbd3*$^{ex1fl/ex1fl}$ MEFs with 4-OHT at 0 hr or 48 hrs after induction of reprogramming factor expression (Figure 3F and Figures S3H and S3I). While both systems demonstrated about 80% downregulation of MBD3 protein, neither impacted on the efficiency of MEF reprogramming. However, depletion of MBD3 protein would take a few days from the time of 4-OHT administration, so it is possible that in this system cells go through the most critical stage of reprogramming with MBD3 protein still present at a sufficient level.

From our experiments we therefore found that, depending on the reprogramming context, MBD3/NuRD depletion can either have no apparent effect on reprogramming or significantly impair the transition to naive pluripotency.

Overexpression of MBD3/NuRD Can Facilitate Reprogramming

Because the complete removal of *Mbd3* or a decrease in its expression can significantly impair the generation of both preiPSCs and iPSCs from NSCs, and iPSCs from preiPSCs and EpiSCs, we tested whether MBD3 levels are limiting for reprogramming. For that, *Oct4*-GFP reporter NSCs and *Nanog*-GFP MEF-derived preiPSCs were stably transfected with *Mbd3* (Figures 4A and 4E). MBD3 overexpression had neither a positive nor detrimental effect on the efficiency of iPSC formation in both systems (Figures 4B, 4C, 4F and 4G). However, combined overexpression of MBD3 and NANOG in MEF-derived preiPSCs led to accelerated reprogramming kinetics and an up to 30-fold increase in reprogramming efficiency compared to *Nanog-Empty* vector (*EV*) control (Figures 4F and 4G and Figures S4A and S4B). This synergistic effect correlated with the upregulation of both *Esrrβ* and endogenous *Oct4* expression, to 5% and 3% of the expression levels of wild-type ESCs, respectively, prior to induction of pluripotency by 2i/LIF medium switch (Figure 4I). Interestingly, MBD3 overexpression in these cell lines caused an increase in protein levels of MTA2, a core subunit of the NuRD

Figure 2. MBD3 Is Required for Efficient iPSC Generation

(A) Quantification of iPSC colonies generated from *Mbd3*$^{fl/-}$, *Mbd3*$^{-/-}$, and *Mbd3*$^{-/-}$*:Mbd3* (rescue) preiPSCs after 2i/LIF culture for 12 days. Colony number is per 1.0×10^5 preiPSCs.

(B) qRT-PCR analysis of retroviral transgenes, *Mbd3*, and pluripotency-associated factors in preiPSCs and corresponding derived iPSCs. qRT-PCR values are normalized to *Gapdh* value and shown as relative to the highest value.

(C) *Mbd3*$^{fl/fl}$*:Cre-ERt2* NSCs were transduced with rMKO and rGFP, maintained in Egf+Fgf2 medium for 3 days, switched to S+LIF for 4 more days to allow preiPSC emergence, and then switched to 2i/LIF conditions to induce iPSC formation. 4-OHT was added at different time points (before or after preiPSC emergence) to induce *Mbd3*-floxed alleles excision. The encircled numbers correspond to different conditions. At day 20 after transfection, GFP$^-$ iPSC colonies were counted and subsequently stained for AP. The number of colonies is presented per 7.5×10^4 NSCs. The error bars indicate STDEV.

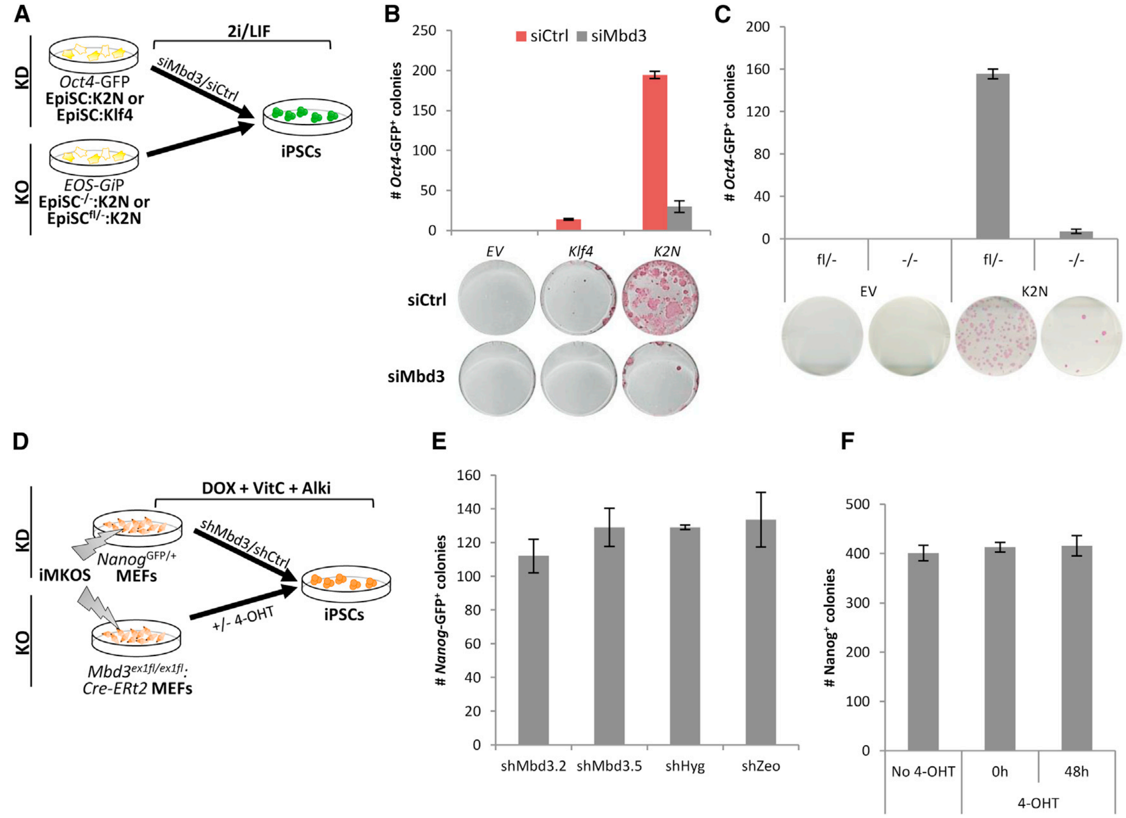

Figure 3. Requirement of MBD3 in Other Reprogramming Systems

(A) Experimental designs used to analyze the effect of *Mbd3* KD and KO on EpiSC reprogramming efficiency. For the KD experiments, wild-type EpiSCs (carrying an *Oct4*-GFP cassette), stably transfected with pPB-CAG-Klf2.2A.Nanog (K2N) or pPB-CAG-Klf4, were transfected with either siMbd3 or siControl (siCtrl) and, after 24 hr, were plated in 2i/LIF for 12 days. For the KO experiments, $Mbd3^{-/-}$ or $Mbd3^{fl-}$ EpiSCs carrying an *Oct4*-GFP reporter (*EOS*-GiP), stably transfected with K2N (or empty vector control, EV), were plated in 2i/LIF for 12 days.

(B and C) The efficiency of EpiSC reprogramming after *Mbd3* removal, either by KD (B) or KO (C), was assessed by counting *Oct4*-GFP^{+} colonies. Representative AP stained plates are also indicated. 1.0 × 10^4 EpiSCs were plated in (B). 1.5 × 10^4 EpiSCs were plated in (C).

(D) Experimental designs used to analyze the effect of *Mbd3* KD and *Mbd3* exon 1 KO. For the KD experiments, *Nanog*-GFP MEFs transfected with doxycycline-inducible MKOS piggyBac transposon (iMKOS) were cultured in S+LIF + DOX + vitamin C (vitC) + Alki 24 hr before lentiviral infections of shMbd3 or shControls. For the KO experiments, $Mbd3^{ex1fl/ex1fl}$ MEFs transfected with iMKOS were infected with pMX-Cre-ERt2. Reprogramming was carried out in S+LIF + DOX + vitC + Alki, and 4-OHT was added either at the time of DOX administration (0h) or 48 hr later (48h).

(E) Number of *Nanog*-GFP^{+} iPSC colonies at day 13 of reprogramming upon infection of indicated shRNAs.

(F) Number of $Nanog^{+}$ colony numbers determined by immunofluorescence after 13 days of reprogramming of $Mbd3^{ex1fl/ex1fl}$:*Cre*-ERt2 MEFs. The error bars indicate STDEV. Typical iMKOS positive cell number at day 2 of reprogramming is 1.0–3.0 × 10^4 cells per well, providing 1%–2% reprogramming efficiency.

complex degraded in the absence of MBD3 (Figures 4D and 4H) (Kaji et al., 2006). This suggests that the effects of MBD3 overexpression are potentially attributable to the total amount, and subsequently total activity, of the NuRD complex. We also found that EpiSCs overexpressing both Nanog and MBD3 showed a 30-fold increase in the ability to generate iPSCs relative to a *Nanog* only control (Figures 4K–4N). Importantly, all iPSCs generated by the overexpression of NANOG and MBD3 exhibited the molecular properties expected for naive pluripotent cells (Figures S4C–S4F) as well as chimera and germline competence after the excision of reprogramming transgenes (Figures 4J and 4O). We did not see reprogramming synergy with the NuRD complex for two other known reprogramming factors, KLF4 and NR5A2 (Figure 4P and Figure S4G).

The MBD3 isoform that we used for the rescue and overexpression experiments was MBD3B, the most abundant isoform by protein levels in ESCs (Figure S4K). In contrast to MBD3B, we found that MBD3C did not synergize with NANOG (Figure 4Q and Figures S4H–S4J). The two isoforms differ in only the first 60 N-terminal amino acids (Figure S4H), indicating that this region is of importance for NANOG-dependent MBD3 ability to facilitate reprogramming.

These results demonstrate that MBD3 overexpression does not impair induction of naive pluripotency and that it can in fact facilitate reprogramming in conjunction with enhanced NANOG expression.

DISCUSSION

In this study we have identified a positive facilitator role for MBD3/NuRD in transcription-factor-mediated reprogramming of NSCs and EpiSCs. In our analyses, we found that genetic

or siRNA-mediated depletion of *Mbd3* led to a reduction in the efficiency of reprogramming in these contexts, but not in reprogramming of MEFs. More specifically, we found through time course experiments that MBD3/NuRD function is particularly important during the initiation phase of reprogramming of NSCs and is more dispensable in the later stages when the pluripotency network is becoming more stably established.

We also found that MBD3 overexpression, with resulting higher levels of the NuRD complex, facilitates reprogramming of MEF-derived preiPSCs and EpiSCs when combined with expression of NANOG, but not with other tested reprogramming factors, which is consistent with previous observations that NuRD complex subunits are high confidence protein interactors of both OCT4 and NANOG (Costa et al., 2013; Ding et al., 2012; Gagliardi et al., 2013; Liang et al., 2008; Pardo et al., 2010; van den Berg et al., 2010). In our experiments the N teminus of the MBD3B isoform, which has previously been suggested to be required for protein-protein interactions (Aguilera et al., 2011), appeared to be required for the observed synergistic effect with NANOG in reprogramming (Figure 4Q). In the future we will aim to understand how NANOG and MBD3 work together to drive cells (preiPSCs) that are arrested in the reprogramming process toward pluripotency.

Our data suggest that the NuRD complex might be facilitating gene activation during reprogramming. Interestingly, MBD3 was recently shown to localize to the regulatory sequences of active genes (Günther et al., 2013; Reynolds et al., 2013; Shimbo et al., 2013), including ESC super-enhancers (Hnisz et al., 2013). Moreover, genome-wide expression analysis revealed that 61% of differentially expressed genes are downregulated after *Mbd3* deletion in ESCs (Reynolds et al., 2012b). Although some of this decrease in transcription might be due to indirect effects, it seems likely that the NuRD complex acts at enhancers as a mediator of transcription-factor-induced gene activation and thus could also interact with pluripotency factors such as NANOG to support genome-wide reprogramming. In addition, NuRD has been proposed to mediate transient mTOR downregulation and subsequent activation of autophagy, a key step during early stages of reprogramming (Wang et al., 2013).

Our results are in apparent disagreement with two recent reports that suggested an inhibitory role for MBD3 in reprogramming (Luo et al., 2013; Rais et al., 2013), including one (Rais et al., 2013) that argued that reduction or deletion of *Mbd3* leads to rapid deterministic reprogramming with 100% efficiency. There are a number of differences between our study and these two previous reports, including the choice of reprogramming cassettes and the reprogramming culture conditions. In contrast to our study, Rais et al. (2013) used a secondary system for somatic cell reprogramming and lentiviral cassette delivery, and it has been reported that both of these factors influence iPSC generation efficiency (Stadtfeld and Hochedlinger, 2010). Moreover, distinct reprogramming factor stoichiometry can provide varying intracellular environments, which may show different dependencies on MBD3 activity for reprogramming. In addition, in our hands heterozygous *Mbd3*$^{fl/-}$ ESCs express MBD3 at nearly wild-type levels (Figures S4K–S4M; Reynolds et al., 2012b), but Rais et al. (2013) reported that their *Mbd3*$^{fl/-}$ ESCs expressed MBD3 at 20% of wild-type levels. Further examination of these and other practical and procedural differences between our study and the previous work should help clarify the basis of the apparent differences seen.

Overall, taking into account the results that we report here and previous studies, our conclusion is that at least in some contexts MBD3/NuRD plays a positive role in reprogramming, and that loss of MBD3 expression leads to a reduction in the efficiency of the reprogramming process.

NuRD plays well-documented roles in controlling gene expression and developmental transitions in a wide variety of different metazoan systems (Ahringer, 2000; McDonel et al., 2009; Reynolds et al., 2013). MBD3 is known to be required for embryonic development and pluripotent cell differentiation (Kaji et al., 2006, 2007), and the composition of the complex or specific interactions of its individual subunits may regulate different aspects of its function (Allen et al., 2013; Reynolds et al., 2013). Further insights into the function of the NuRD complex during different cell state transitions will help us understand the process of induced pluripotency as well as embryonic development.

EXPERIMENTAL PROCEDURES

Cell Culture

Platinum-E, preiPSCs, and MEFs were cultured in GMEM (Sigma-Aldrich) supplemented with 10% FCS, 1× NEAA, 1× Pen/Strep, 1 mM sodium pyruvate, 0.1 mM 2-mercaptoethanol, 2 mM L-glutamine, and 20 ng/ml of LIF (homemade), indicated as *S*+LIF medium throughout. ESCs and iPSCs were maintained in N2B27-based medium (DMEM/F12 and Neurobasal [both Life Technologies] in 1:1 ratio, 1× Pen/Strep, 0.1 mM 2-mercaptoethanol, 2 mM L-glutamine, 1:200 N2 [PAA], and 1:100 B27 [Life Technologies]) supplemented 20 ng/ml of LIF and 2i inhibitors: CHIR99021 (3 μM) and PD0325901 (1 μM), indicated as 2i/LIF throughout (Ying et al., 2008). NSCs were cultured in DMEM/F12 (GIBCO) supplemented with 1× NEAA, 0.1 mM 2-mercaptoethanol, 1× Pen/Strep, 1:100 B27, 1:200 N2 supplement, 4.5 μM HEPES, 0.03 M glucose, 120 μg/ml BSA, 10 ng/ml of Egf (Peprotech), and 20 ng/ml of Fgf2 (homemade), indicated as Egf+Fgf2 medium throughout. EpiSCs were maintained in N2B27-based medium containing 12 ng/ml of Fgf2 and 20 ng/ml of Activin A (homemade), indicated as Fgf2/Act.A medium throughout. EpiSCs and NSCs were cultured on plastic coated with fibronectin (10 μg/ml, Millipore) or laminin (10 μg/ml, Sigma-Aldrich), respectively. All other cell types were grown on gelatine. All cell types were maintained at 7% CO_2. For Cre-mediated transgene excision, cells were treated with 500 nM of 4-OHT.

Derivation of Cell Lines

NSCs

Brains from *Mbd3*$^{fl/fl}$ and *Mbd3*$^{ex1fl/ex1fl}$ E13.5 embryos were dissected, dissociated in Egf+Fgf2 medium, and plated onto the laminin-coated cell culture flasks. *Mbd3*$^{fl/-}$ NSCs were derived from ESCs as described (Pollard et al., 2006). Briefly, ESCs were seeded on gelatinized 10 cm dishes in N2B27 medium for 7 days. After this period, cells were trypsinized and plated on nongelatinized dishes for 3 days in Egf+Fgf2 medium. The emergent neurospheres were then seeded on gelatinized plates and maintained in monolayer in Egf+Fgf2 medium. For Cre-excision of the *Mbd3* floxed allele, *Mbd3*$^{fl/-}$ NSCs were nucleofected with a pCAG-Cre-ires-Puro plasmid and clonal lines of *Mbd3*$^{-/-}$ NSCs were expanded.

MEFs

Organ-deprived carcasses from E12.5 or E13.5 embryos were dissociated into small pieces, trypsinized, and plated in S+LIF medium.

EpiSCs

Mbd3$^{fl/-}$ and *Mbd3*$^{-/-}$ EpiSCs were derived from ESCs as previously described (Guo et al., 2009). Briefly, ESCs transfected with pPB-EOS-GFP-ires-Puro (*EOS*-GiP; GFPiresPuro under the control of early transposon promoter and *Oct4* and *Sox2* enhancers) were cultured in Fgf2/Act.A medium

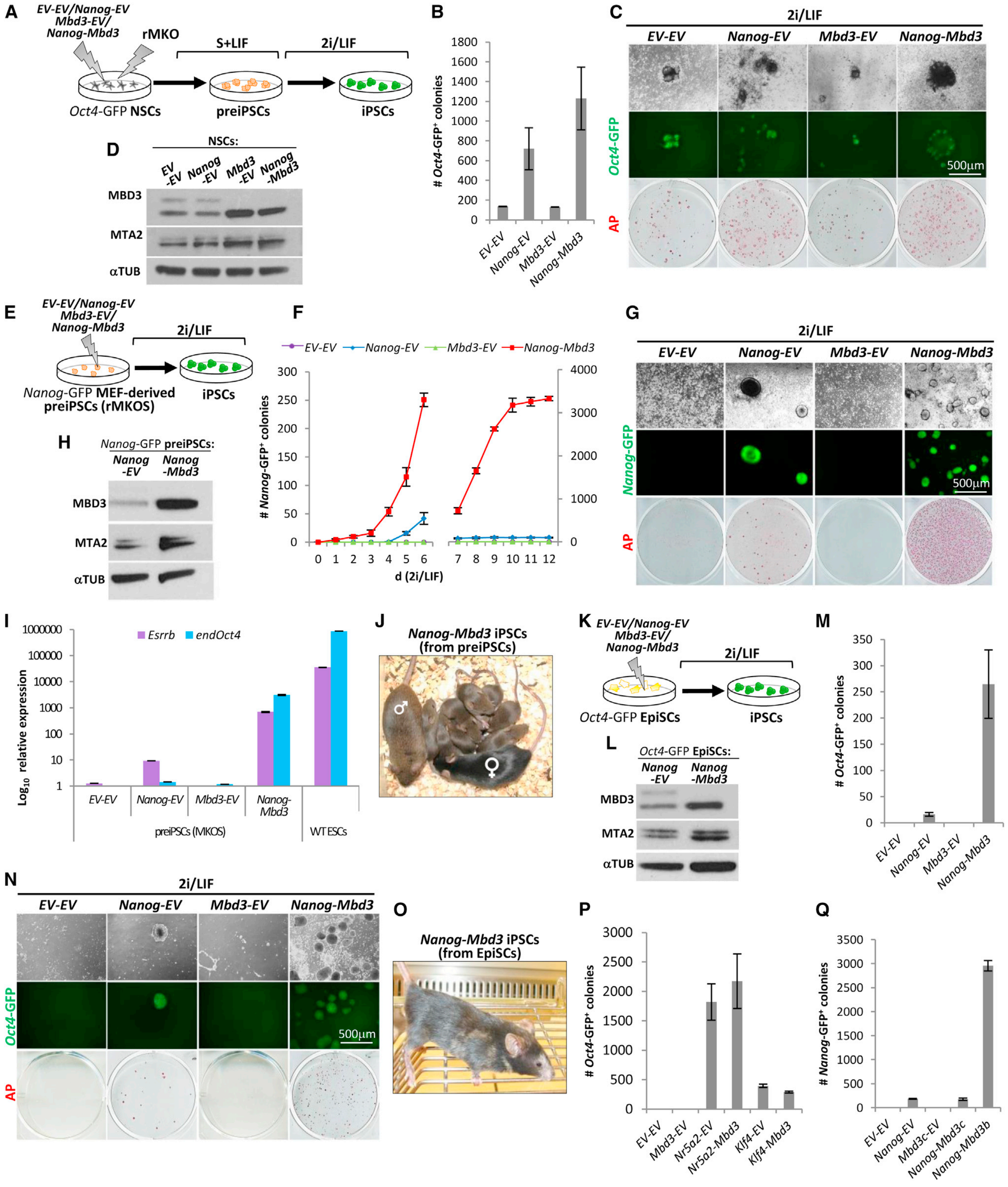

Figure 4. Overexpression of MBD3/NuRD Facilitates NANOG-Mediated Reprogramming

(A) Experimental design used to address the effect of MBD3 overexpression on NSC reprogramming. NSCs carrying an *Oct4*-GFP cassette were stably transfected with pPB-CAG-Nanog and pPB-CAG-Mbd3b or pPB-CAG-empty controls, transduced with rOKM, cultured in Egf+Fgf2 medium for 3 days, switched to S+LIF medium for 6 days, and then switched to 2i/LIF conditions.

(B) Quantification of *Oct4*-GFP$^+$ colonies after 12 days in 2i/LIF conditions. Colony number is per 1.0 × 10^5 NSCs.

(C) Phase and GFP images and AP staining of the iPSCs obtained from NSCs overexpressing respective transgenes.

(legend continued on next page)

for at least 10 passages before analysis. To obtain a pure EpiSC culture, GFP+ cells were removed by FACS.

SUPPLEMENTAL INFORMATION

Supplemental Information for this article includes four figures and Supplemental Experimental Procedures and can be found with this article online at http://dx.doi.org/10.1016/j.stem.2014.04.019.

ACKNOWLEDGMENTS

We acknowledge N. Reynolds and J. Ramalho-Santos for advice and helpful discussions. We are also grateful to Y. Costa for critical reading of the manuscript. This study was supported by a Wellcome Trust Fellowship (WT101861), an ERC starting grant, and the Anne Rowling Clinic. J.C.R.S. and B.H. are Wellcome Trust Senior Research Fellows in the Basic Biomedical Sciences. R.L.S. is a recipient of a Ph.D. fellowship from the Portuguese Foundation for Sciences and Technology, FCT (SFRH/BD/51198/2010). L.T. is a recipient of a Ph.D. fellowship from The College of Medicine and Veterinary Medicine, University of Edinburgh.

Received: January 8, 2014
Revised: March 27, 2014
Accepted: April 24, 2014
Published: May 15, 2014

REFERENCES

Aguilera, C., Nakagawa, K., Sancho, R., Chakraborty, A., Hendrich, B., and Behrens, A. (2011). c-Jun N-terminal phosphorylation antagonises recruitment of the Mbd3/NuRD repressor complex. Nature *469*, 231–235.

Ahringer, J. (2000). NuRD and SIN3 histone deacetylase complexes in development. Trends Genet. *16*, 351–356.

Allen, H.F., Wade, P.A., and Kutateladze, T.G. (2013). The NuRD architecture. Cell. Mol. Life Sci. *70*, 3513–3524.

Apostolou, E., and Hochedlinger, K. (2013). Chromatin dynamics during cellular reprogramming. Nature *502*, 462–471.

Costa, Y., Ding, J., Theunissen, T.W., Faiola, F., Hore, T.A., Shliaha, P.V., Fidalgo, M., Saunders, A., Lawrence, M., Dietmann, S., et al. (2013). NANOG-dependent function of TET1 and TET2 in establishment of pluripotency. Nature *495*, 370–374.

Ding, J., Xu, H., Faiola, F., Ma'ayan, A., and Wang, J. (2012). Oct4 links multiple epigenetic pathways to the pluripotency network. Cell Res. *22*, 155–167.

Gagliardi, A., Mullin, N.P., Ying Tan, Z., Colby, D., Kousa, A.I., Halbritter, F., Weiss, J.T., Felker, A., Bezstarosti, K., Favaro, R., et al. (2013). A direct physical interaction between Nanog and Sox2 regulates embryonic stem cell self-renewal. EMBO J. *32*, 2231–2247.

Günther, K., Rust, M., Leers, J., Boettger, T., Scharfe, M., Jarek, M., Bartkuhn, M., and Renkawitz, R. (2013). Differential roles for MBD2 and MBD3 at methylated CpG islands, active promoters and binding to exon sequences. Nucleic Acids Res. *41*, 3010–3021.

Guo, G., Yang, J., Nichols, J., Hall, J.S., Eyres, I., Mansfield, W., and Smith, A. (2009). Klf4 reverts developmentally programmed restriction of ground state pluripotency. Development *136*, 1063–1069.

Hendrich, B., Guy, J., Ramsahoye, B., Wilson, V.A., and Bird, A. (2001). Closely related proteins MBD2 and MBD3 play distinctive but interacting roles in mouse development. Genes Dev. *15*, 710–723.

Hnisz, D., Abraham, B.J., Lee, T.I., Lau, A., Saint-André, V., Sigova, A.A., Hoke, H.A., and Young, R.A. (2013). Super-enhancers in the control of cell identity and disease. Cell *155*, 934–947.

Kaji, K., Caballero, I.M., MacLeod, R., Nichols, J., Wilson, V.A., and Hendrich, B. (2006). The NuRD component Mbd3 is required for pluripotency of embryonic stem cells. Nat. Cell Biol. *8*, 285–292.

Kaji, K., Nichols, J., and Hendrich, B. (2007). Mbd3, a component of the NuRD co-repressor complex, is required for development of pluripotent cells. Development *134*, 1123–1132.

Kaji, K., Norrby, K., Paca, A., Mileikovsky, M., Mohseni, P., and Woltjen, K. (2009). Virus-free induction of pluripotency and subsequent excision of reprogramming factors. Nature *458*, 771–775.

Kim, J.B., Sebastiano, V., Wu, G., Araúzo-Bravo, M.J., Sasse, P., Gentile, L., Ko, K., Ruau, D., Ehrich, M., van den Boom, D., et al. (2009). Oct4-induced pluripotency in adult neural stem cells. Cell *136*, 411–419.

Lai, A.Y., and Wade, P.A. (2011). Cancer biology and NuRD: a multifaceted chromatin remodelling complex. Nat. Rev. Cancer *11*, 588–596.

Liang, J., Wan, M., Zhang, Y., Gu, P.L., Xin, H.W., Jung, S.Y., Qin, J., Wong, J.M., Cooney, A.J., Liu, D., and Songyang, Z. (2008). Nanog and Oct4

(D) Western blot analysis of MBD3, MTA2, and TUBULIN (TUB) protein expression in NSCs overexpressing the indicated transgene combinations.
(E) Experimental design used to address the effect of MBD3/NuRD overexpression on the conversion of preiPSCs to iPSCs. PreiPSCs (carrying a *Nanog*-GFP) were stably transfected with the same transgene combinations as in (A) and plated in 2i/LIF conditions for 12 days.
(F) The kinetics of the emergence of *Nanog*-GFP+ colonies from the transgenic preiPSCs during a 12 day culture in 2i/LIF conditions (y axis scale changes at day 7). Colony number is per 1.0×10^5 preiPSCs.
(G) Phase and GFP images and AP staining of the iPSCs formed from preiPSCs overexpressing respective transgenes.
(H) Western blot analysis of MBD3, MTA2, and TUBULIN (TUB) protein expression in preiPSCs overexpressing NANOG or NANOG and MBD3.
(I) qRT-PCR analysis of *Esrrβ* and endogenous (end) *Oct4* expression in preiPSCs 12 days after stable transgene transfection and culture in S+LIF (y axes in $\log_{10}$ scale). The expression levels of *Esrrβ* and endogenous *Oct4* in these *Nanog-Mbd3* preiPSCs are 5% and 3%, respectively, of the expression levels of WT ESCs in 2i/LIF. The *Esrrβ* expression level is also approximately 80 times greater than that of *Nanog-EV* preiPSCs and 700 times greater than that of *Mbd3-EV* and *EV-EV* preiPSCs. The endogenous *Oct4* expression level is approximately 2,000 times greater than that of *Nanog-EV* preiPSCs and 3,000 times greater than that of *Mbd3-EV* and *EV-EV* preiPSCs. qRT-PCR values are normalized to *Gapdh* value and shown as relative to the highest value.
(J) Germ line contribution of *Nanog-Mbd3* iPSCs generated from preiPSCs (brown color). Cells were treated with TAT-Cre for reprogramming transgene excision prior to blastocyst injection. Chimeric father, C57BL/6 mother, and pups resulting from cross can be viewed.
(K) Experimental design used to address the effect of MBD3/NuRD overexpression on EpiSC reprogramming. EpiSCs (carrying an *Oct4*-GFP) were stably transfected with the same transgene combinations as in (A) and (E) and plated in 2i/LIF conditions for 12 days.
(L) Western blot analysis of MBD3, MTA2, and TUBULIN (TUB) protein expression in EpiSCs overexpressing NANOG or NANOG and MBD3.
(M) Quantification of *Oct4*-GFP+ colonies after 12 days of 2i/LIF culture. Colony numbers are per 2.0×10^4 EpiSCs.
(N) Phase and GFP images and AP staining of the iPSCs formed from EpiSCs overexpressing respective transgenes.
(O) Chimera of *Nanog-Mbd3* iPSCs generated from EpiSCs (brown color). Cells were treated with TAT-Cre for reprogramming transgene excision prior to blastocyst injection.
(P) Quantification of *Oct4*-GFP+ colonies after 12 days of 2i/LIF culture, generated from EpiSCs transfected with *Klf4* or *Nr5a2* (together or not with *Mbd3*). Colony numbers are per 2.0×10^4 EpiSCs.
(Q) Quantification of *Nanog*-GFP+ colonies after 12 days of 2i/LIF culture generated from MEF-derived preiPSCs stably transfected with *Nanog* alone, or *Nanog* together with *Mbd3b* or *Mbd3c*. Colony numbers are per 1.0×10^5 preiPSCs. The error bars indicate STDEV.

associate with unique transcriptional repression complexes in embryonic stem cells. Nat. Cell Biol. *10*, 731–739.

Luo, M., Ling, T., Xie, W., Sun, H., Zhou, Y., Zhu, Q., Shen, M., Zong, L., Lyu, G., Zhao, Y., et al. (2013). NuRD blocks reprogramming of mouse somatic cells into pluripotent stem cells. Stem Cells *31*, 1278–1286.

McDonel, P., Costello, I., and Hendrich, B. (2009). Keeping things quiet: roles of NuRD and Sin3 co-repressor complexes during mammalian development. Int. J. Biochem. Cell Biol. *41*, 108–116.

Papp, B., and Plath, K. (2013). Epigenetics of reprogramming to induced pluripotency. Cell *152*, 1324–1343.

Pardo, M., Lang, B., Yu, L., Prosser, H., Bradley, A., Babu, M.M., and Choudhary, J. (2010). An expanded Oct4 interaction network: implications for stem cell biology, development, and disease. Cell Stem Cell *6*, 382–395.

Pollard, S.M., Benchoua, A., and Lowell, S. (2006). Neural stem cells, neurons, and glia. In Embryonic Stem Cells, I.L.R. Klimanskaya, ed. (Boston: Elsevier Academic Press), pp. 151–169.

Radzisheuskaya, A., and Silva, J.C. (2014). Do all roads lead to Oct4? The emerging concepts of induced pluripotency. Trends Cell Biol. *24*, 275–284.

Radzisheuskaya, A., Chia, Gle.B., dos Santos, R.L., Theunissen, T.W., Castro, L.F., Nichols, J., and Silva, J.C. (2013). A defined Oct4 level governs cell state transitions of pluripotency entry and differentiation into all embryonic lineages. Nat. Cell Biol. *15*, 579–590.

Rais, Y., Zviran, A., Geula, S., Gafni, O., Chomsky, E., Viukov, S., Mansour, A.A., Caspi, I., Krupalnik, V., Zerbib, M., et al. (2013). Deterministic direct reprogramming of somatic cells to pluripotency. Nature *502*, 65–70.

Reynolds, N., Latos, P., Hynes-Allen, A., Loos, R., Leaford, D., O'Shaughnessy, A., Mosaku, O., Signolet, J., Brennecke, P., Kalkan, T., et al. (2012a). NuRD suppresses pluripotency gene expression to promote transcriptional heterogeneity and lineage commitment. Cell Stem Cell *10*, 583–594.

Reynolds, N., Salmon-Divon, M., Dvinge, H., Hynes-Allen, A., Balasooriya, G., Leaford, D., Behrens, A., Bertone, P., and Hendrich, B. (2012b). NuRD-mediated deacetylation of H3K27 facilitates recruitment of Polycomb Repressive Complex 2 to direct gene repression. EMBO J. *31*, 593–605.

Reynolds, N., O'Shaughnessy, A., and Hendrich, B. (2013). Transcriptional repressors: multifaceted regulators of gene expression. Development *140*, 505–512.

Shimbo, T., Du, Y., Grimm, S.A., Dhasarathy, A., Mav, D., Shah, R.R., Shi, H., and Wade, P.A. (2013). MBD3 localizes at promoters, gene bodies and enhancers of active genes. PLoS Genet. *9*, e1004028.

Silva, J., Barrandon, O., Nichols, J., Kawaguchi, J., Theunissen, T.W., and Smith, A. (2008). Promotion of reprogramming to ground state pluripotency by signal inhibition. PLoS Biol. *6*, e253.

Silva, J., Nichols, J., Theunissen, T.W., Guo, G., van Oosten, A.L., Barrandon, O., Wray, J., Yamanaka, S., Chambers, I., and Smith, A. (2009). Nanog is the gateway to the pluripotent ground state. Cell *138*, 722–737.

Sims, J.K., and Wade, P.A. (2011). Mi-2/NuRD complex function is required for normal S phase progression and assembly of pericentric heterochromatin. Mol. Biol. Cell *22*, 3094–3102.

Sommer, C.A., Stadtfeld, M., Murphy, G.J., Hochedlinger, K., Kotton, D.N., and Mostoslavsky, G. (2009). Induced pluripotent stem cell generation using a single lentiviral stem cell cassette. Stem Cells *27*, 543–549.

Stadtfeld, M., and Hochedlinger, K. (2010). Induced pluripotency: history, mechanisms, and applications. Genes Dev. *24*, 2239–2263.

van den Berg, D.L.C., Snoek, T., Mullin, N.P., Yates, A., Bezstarosti, K., Demmers, J., Chambers, I., and Poot, R.A. (2010). An Oct4-centered protein interaction network in embryonic stem cells. Cell Stem Cell *6*, 369–381.

Wang, S., Xia, P., Ye, B., Huang, G., Liu, J., and Fan, Z. (2013). Transient activation of autophagy via Sox2-mediated suppression of mTOR is an important early step in reprogramming to pluripotency. Cell Stem Cell *13*, 617–625.

Ying, Q.L., Wray, J., Nichols, J., Batlle-Morera, L., Doble, B., Woodgett, J., Cohen, P., and Smith, A. (2008). The ground state of embryonic stem cell self-renewal. Nature *453*, 519–523.

Zhang, Y., Ng, H.H., Erdjument-Bromage, H., Tempst, P., Bird, A., and Reinberg, D. (1999). Analysis of the NuRD subunits reveals a histone deacetylase core complex and a connection with DNA methylation. Genes Dev. *13*, 1924–1935.

Cell Stem Cell, Volume *15*

Supplemental Information

MBD3/NuRD Facilitates Induction of Pluripotency in a Context-Dependent Manner

Rodrigo L. dos Santos, Luca Tosti, Aliaksandra Radzisheuskaya, Isabel M. Caballero, Keisuke Kaji, Brian Hendrich, and José C. R. Silva

Supplemental Figures 1-4

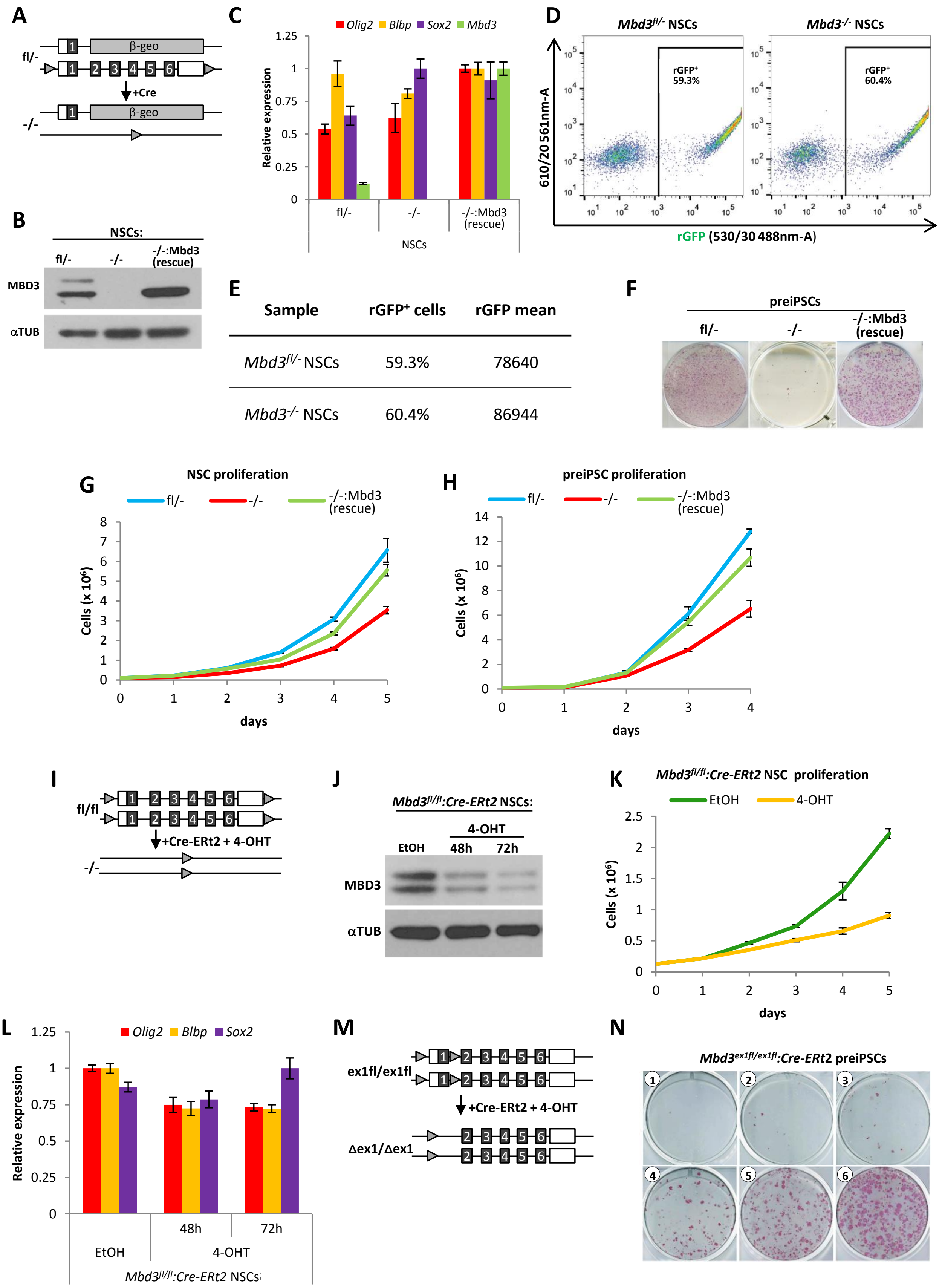

Figure S1, related to Figure 1 - Mbd3 facilitates the initiation of reprogramming.

(A) Schematic representation of *Mbd3* loci in NSCs *Mbd3*$^{fl/-}$ and *Mbd3*$^{-/-}$. Exons are indicated as dark grey boxes, non-coding sequences are indicated as unfilled boxes, and light grey triangles represent loxP sites. **(B)** Western blot analysis of MBD3 and α-TUBULIN (αTUB) protein levels in the *Mbd3*$^{fl/-}$, *Mbd3*$^{-/-}$ and *Mbd3*$^{-/-:}$*Mbd3* (rescue) NSCs used for reprogramming experiments in Figures 1A-C. **(C)** qRT-PCR analysis of NSC markers (*Sox2*, *Olig2*, *Blbp*) and *Mbd3* expression levels in NSCs. **(D)** Retroviral GFP (rGFP) expression 72h after transduction of *Mbd3*$^{fl/-}$ or *Mbd3*$^{-/-}$ NSCs with pMX-GFP, assessed by flow cytometry. GFP^{+} gates are shown. **(E)** Table indicates the percentage of rGFP^{+} cells and rGFP mean 72h after transduction of *Mbd3*$^{fl/-}$ or *Mbd3*$^{-/-}$ NSCs with pMX-GFP. **(F)** Alkaline phosphatase (AP) staining of preiPSCs 9 days post-transduction of NSCs *Mbd3*$^{fl/-}$, *Mbd3*$^{-/-}$ and rescue NSC lines. **(G)** Cell proliferation analysis of *Mbd3*$^{fl/-}$*, Mbd3*$^{-/-}$ and rescue NSC lines. **(H)** Cell proliferation analysis of *Mbd3*$^{fl/-}$*, Mbd3*$^{-/-}$ and *Mbd3*$^{-/-}$*:Mbd3* preiPSCs lines. **(I)** Schematic representation of *Mbd3* loci in *Mbd3*$^{fl/fl}$ NSCs before and after transfection with the pCAG-CreERt2 transgene and treatment with 4-OHT. Exons are indicated as dark grey boxes, non-coding sequences are indicated as unfilled boxes, and light grey triangles represent loxP sites. **(J)** Western blot analysis of MBD3 and α-TUBULIN (αTUB) protein levels in the *Mbd3*$^{fl/fl}$*:Cre-ERt2* NSCs treated with tamoxifen (4-OHT) or ethanol (EtOH). **(K)** Cell proliferation analysis of NSCs *Mbd3*$^{fl/fl}$*:Cre-ERt2*, in the presence of 4-OHT or EtOH. **(L)** qRT-PCR analysis of NSC markers expression in *Mbd3*$^{fl/fl}$*:Cre-ERt2* NSCs treated with 4-OHT or EtOH. qRT-PCR values are normalized to *Gapdh* value and shown as relative to the highest value. The error bars indicate STDEV. **(M)** Schematic representation of *Mbd3* loci in *Mbd3*$^{ex1fl/ex1fl}$ NSCs before and after transfection with pCAG-Cre-ERt2 transgene and treatment with 4-OHT. Exons are indicated as dark grey boxes, non-coding sequences are indicated as unfilled boxes, and

light grey triangles represent loxP sites. **(N)** Time-course of the removal of *Mbd3* exon 1 during initiation of reprogramming. *Mbd3*$^{ex1fl/ex1fl}$ NSCs were stably transfected with the pCAG-Cre-ERt2 transgene, transduced with retroviruses expressing reprogramming factors and treated with 4-OHT at different reprogramming time points to induce Cre-mediated deletion of the floxed alleles. EtOH was used as a control. The efficiency of preiPSC formation was assessed by AP staining at day 10 post transduction. The encircled numbers correspond to different conditions. For schematic representation of different conditions and understanding of labelling, refer to Figure 1E.

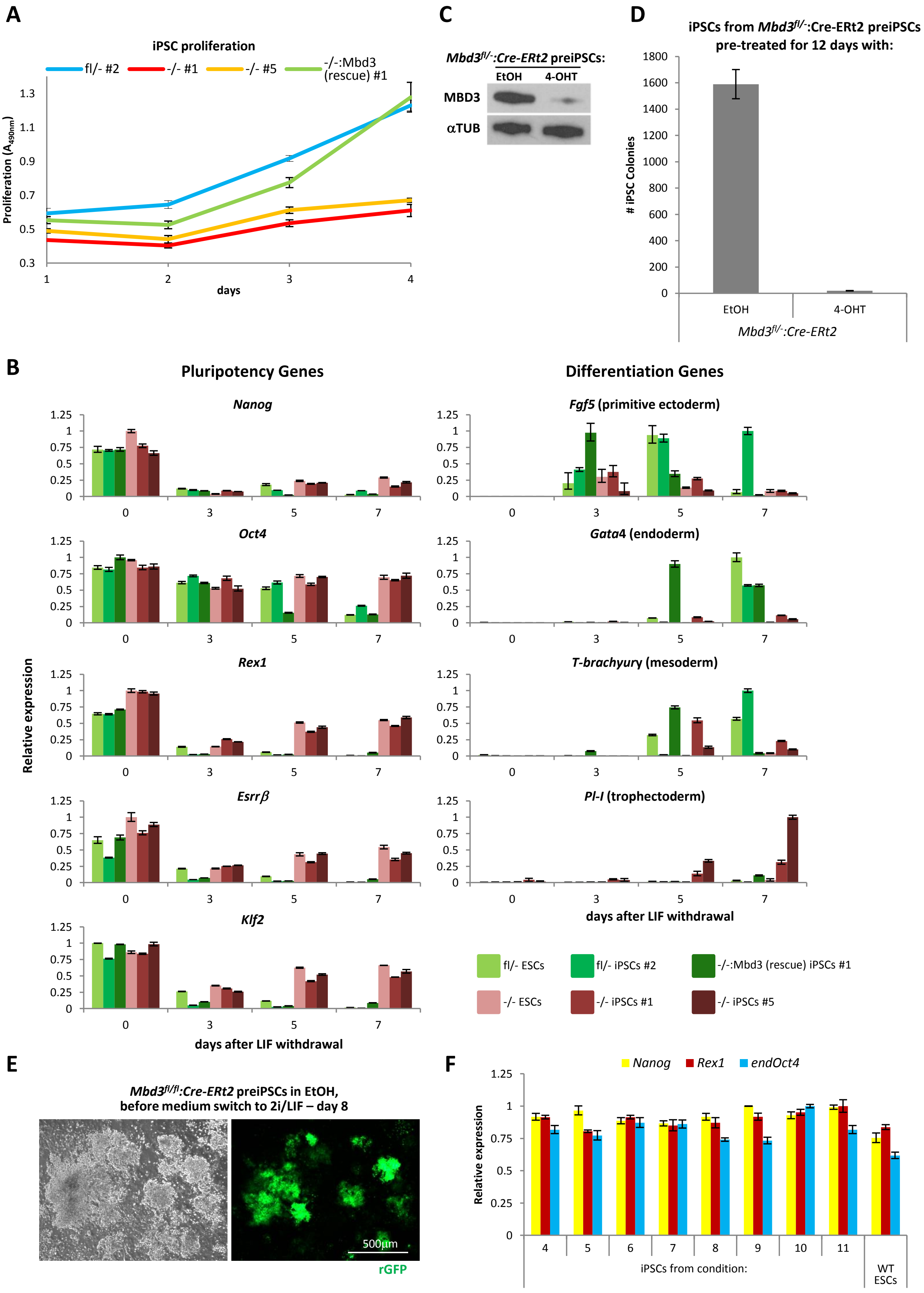
A
iPSC proliferation
fl/- #2
-/- #1
-/- #5
-/-:Mbd3 (rescue) #1
Proliferation (A_{490nm})
days
C
Mbd3fl/-:Cre-ERt2 preiPSCs:
EtOH
4-OHT
MBD3
αTUB
D
iPSCs from Mbd3fl/-:Cre-ERt2 preiPSCs pre-treated for 12 days with:
iPSC Colonies
EtOH
4-OHT
Mbd3fl/-:Cre-ERt2
B
Pluripotency Genes
Differentiation Genes
Nanog
Oct4
Rex1
Esrrβ
Klf2
Fgf5 (primitive ectoderm)
Gata4 (endoderm)
T-brachyury (mesoderm)
Pl-I (trophectoderm)
Relative expression
days after LIF withdrawal
fl/- ESCs
fl/- iPSCs #2
-/-:Mbd3 (rescue) iPSCs #1
-/- ESCs
-/- iPSCs #1
-/- iPSCs #5
E
Mbd3fl/fl:Cre-ERt2 preiPSCs in EtOH, before medium switch to 2i/LIF – day 8
500μm
rGFP
F
Nanog
Rex1
endOct4
Relative expression
iPSCs from condition:
WT ESCs

Figure S2, related to Figure 2 - Mbd3 is required for efficient iPSC generation.

(A) Cell proliferation analysis of *Mbd3*$^{fl/-}$, *Mbd3*$^{-/-}$ and *Mbd3*$^{-/-}$*:Mbd3* iPSCs culture in 2i/LIF conditions. **(B)** qRT-PCR analysis of pluripotency- and differentiation-associated genes during differentiation as embryoid bodies of *Mbd3*$^{fl/-}$, *Mbd3*$^{-/-}$, *Mbd3*$^{-/-}$*:Mbd3* iPSCs and ESCs controls. **(C)** Western blot analysis of MBD3 and α-TUBULIN (αTUB) protein levels in the *Mbd3*$^{fl/-}$*:Cre-ERt2* preiPSC treated with 4-OHT or EtOH for 12 days. **(D)** Quantification of iPSCs colonies generated from *Mbd3*$^{fl/-}$ preiPSCs stably transformed with pCAG-Cre-ERt2 and pre-treated with 4-OHT or EtOH for 12 days while cultured in S+LIF. Medium was switched from S+LIF to 2i/LIF 24h after plating. No 4-OHT or EtOH was added during 2i/LIF culture. Colony number is per 1.0 x 10^5 preiPSCs plated. **(E)** Phase and GFP images of *Mbd3*$^{fl/fl}$*:Cre-ERt2* preiPSC colonies 8 days after transduction with rOKM + rGFP in presence of EtOH. Images taken before medium switch to 2i/LIF, day 8. **(F)** qRT-PCR analysis of pluripotency-associated genes in iPSCs generated from *Mbd3*$^{fl/fl}$*:Cre-ERt2* NSCs with *Mbd3* deletion at different time points of reprogramming, as described in Figure 2C. qRT-PCR values are normalized to *Gapdh* value and shown as relative to the highest value. The error bars indicate STDEV.

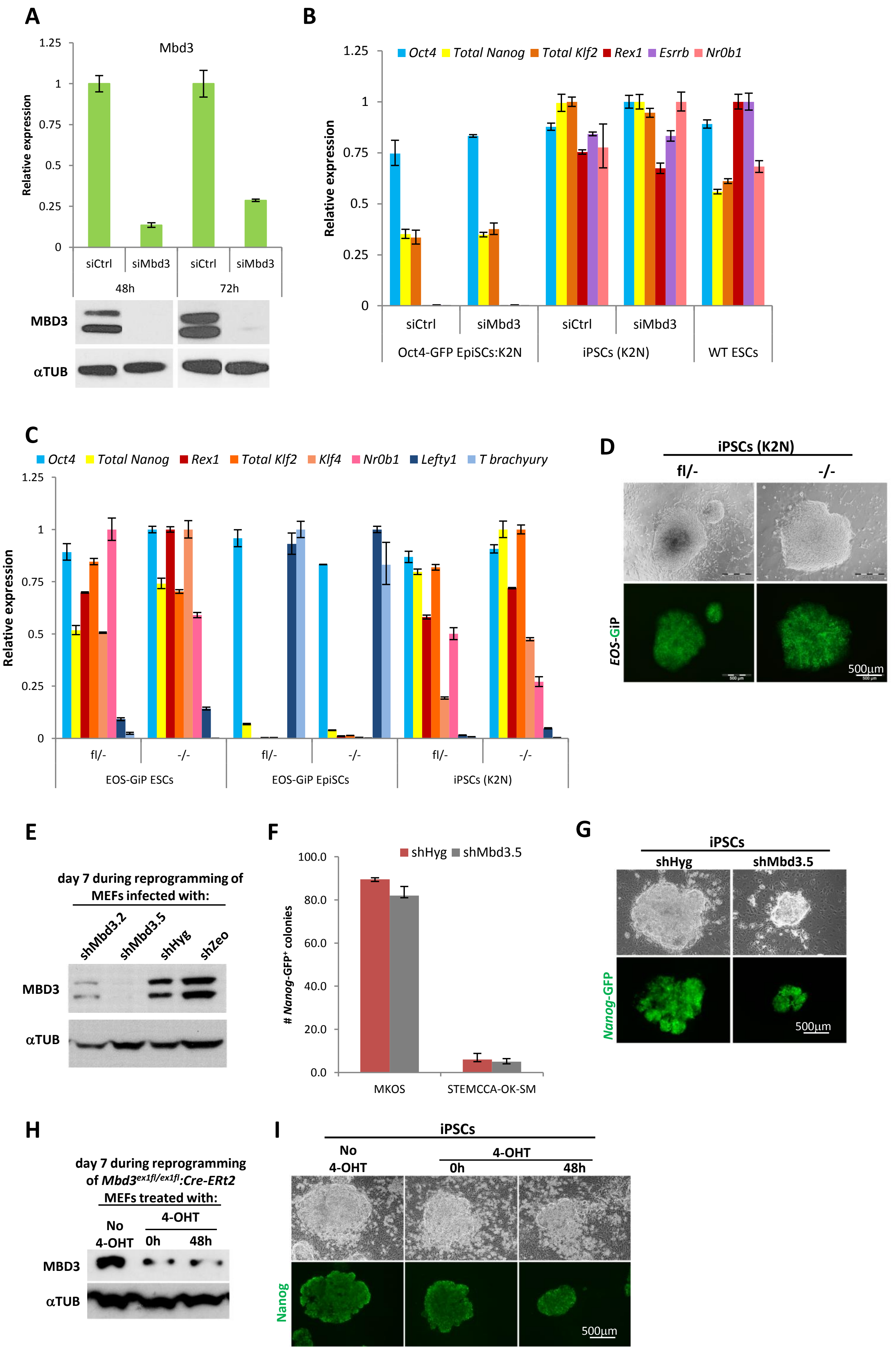
A
Mbd3
Relative expression
1.25
1
0.75
0.5
0.25
0
siCtrl
siMbd3
siCtrl
siMbd3
48h
72h
MBD3
αTUB
B
Oct4
Total Nanog
Total Klf2
Rex1
Esrrb
Nr0b1
Relative expression
siCtrl
siMbd3
siCtrl
siMbd3
Oct4-GFP EpiSCs:K2N
iPSCs (K2N)
WT ESCs
C
Oct4
Total Nanog
Rex1
Total Klf2
Klf4
Nr0b1
Lefty1
T brachyury
Relative expression
fl/-
-/-
fl/-
-/-
fl/-
-/-
EOS-GiP ESCs
EOS-GiP EpiSCs
iPSCs (K2N)
D
iPSCs (K2N)
fl/-
-/-
EOS-GiP
500μm
E
day 7 during reprogramming of
MEFs infected with:
shMbd3.2
shMbd3.5
shHyg
shZeo
MBD3
αTUB
F
shHyg
shMbd3.5
Nanog-GFP+ colonies
100.0
80.0
60.0
40.0
20.0
0.0
MKOS
STEMCCA-OK-SM
G
iPSCs
shHyg
shMbd3.5
Nanog-GFP
500μm
H
day 7 during reprogramming
of Mbd3ex1fl/ex1fl:Cre-ERt2
MEFs treated with:
No
4-OHT
4-OHT
0h
48h
MBD3
αTUB
I
iPSCs
No
4-OHT
4-OHT
0h
48h
Nanog
500μm

Figure S3, related to Figure 3 - Requirement of Mbd3 in other reprogramming systems

(A) qRT-PCR and western blot analysis of *Mbd3* transcript and MBD3 and α-TUBULIN (αTUB) protein levels, respectively, 48h and 72h after siRNA-mediated KD. **(B)** qRT-PCR analysis of *Oct4*, total *Nanog*, *Rex1*, *Esrrβ*, total *Klf2* and *Nr0b1* expression in Klf2-2A-Nanog transfected *Oct4*-GFP EpiSCs and derived from them iPSCs after siCtrl and siMbd3 transfection in 2i/LIF. **(C)** qRT-PCR analysis of *Oct4*, total *Nanog*, *Rex1*, total *Klf2*, *Klf4*, *Nr0b1*, *Lefty1* and *T (brachyury)* expression in parental *Mbd3*$^{fl/-}$ and *Mbd3*$^{-/-}$ *EOS*-GiP ESCs, *Mbd3*$^{fl/-}$ and *Mbd3*$^{-/-}$ *EOS*-GiP EpiSCs obtained from them, and EpiSC-derived iPSCs. qRT-PCR values are normalized to *Gapdh* value and shown as relative to the highest value. **(D)** Phase and *EOS*-GiP (GFPiresPuro under the control of early transposon promoter and Oct4 and Sox2 enhancers) images of *Mbd3*$^{fl/-}$ and *Mbd3*$^{-/-}$ iPSCs generated from EpiSCs. **(E)** Western blot analysis of MBD3 and α-TUBULIN (αTUB) protein levels at day 7 of reprogramming of MEFs infected with shMbd3 or control shRNA against Hygromycin or Zeocin resistant genes (shHyg or shZeo). Over 90% of MBD3 knockdown was observed. **(F)** Quantification of *Nanog*-GFP^{+} iPSCs colonies generated by piggyBac MEF reprogramming using two different reprogramming cassettes, MKOS and STEMCCA-OK-SM. *Mbd3* KD using shRNA was carried out 24h after induction of the reprogramming cassettes. The error bars indicate STDEV. **(G)** Phase and *Nanog*-GFP images of iPSCs derived from MEFs transduced with lentiviruses encoding shHyg or shMbd3.5. **(H)** Western blot analysis of MBD3 and α-TUBULIN (αTUB) protein levels at day 7 during reprogramming of *Mbd3*$^{ex1fl/ex1fl:}$*Cre-ERt2* MEFs treated with 4-OHT 0h or 48h after induction of the reprogramming cassettes. About 80% of MBD3 knockdown was observed. **(I)** Phase and Nanog immunofluorescence images of the Nanog-positive iPSC colonies derived from *Mbd3*$^{ex1fl/ex1fl:}$*Cre-ERt2* treated with 4-OHT 0h or 48h after induction of the reprogramming cassettes.

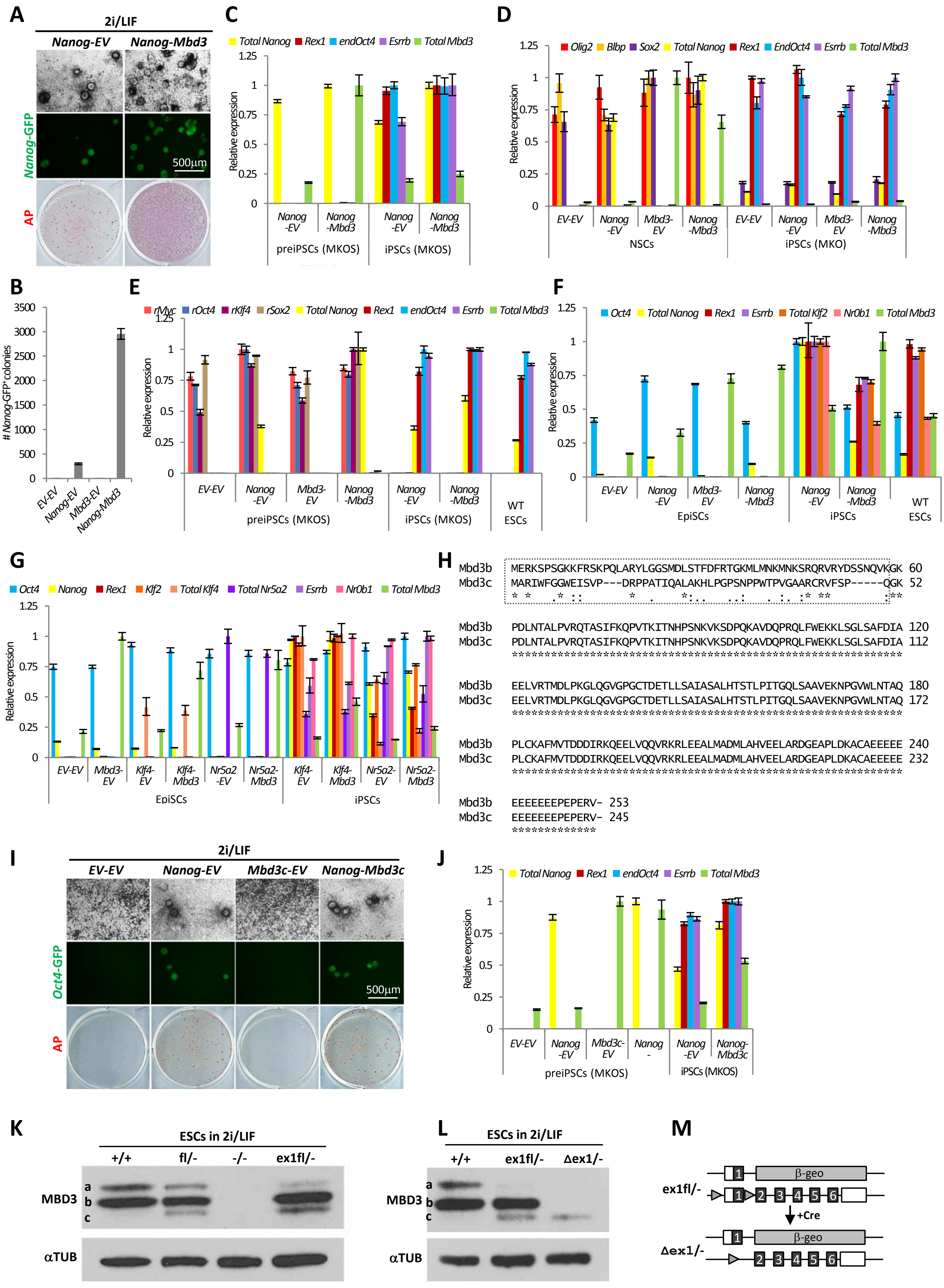
A
2i/LIF
Nanog-EV
Nanog-Mbd3
Nanog-GFP
500µm
AP
B
Nanog-GFP+ colonies
EV-EV
Nanog-EV
Mbd3-EV
Nanog-Mbd3
C
Total Nanog
Rex1
endOct4
Esrrb
Total Mbd3
Relative expression
preiPSCs (MKOS)
iPSCs (MKOS)
D
Olig2
Blbp
Sox2
Total Nanog
Rex1
EndOct4
Esrrb
Total Mbd3
NSCs
iPSCs (MKO)
E
rMyc
rOct4
rKlf4
rSox2
Total Nanog
Rex1
endOct4
Esrrb
Total Mbd3
WT ESCs
F
Oct4
Total Nanog
Rex1
Esrrb
Total Klf2
Nr0b1
Total Mbd3
EpiSCs
iPSCs
WT ESCs
G
Oct4
Nanog
Rex1
Klf2
Total Klf4
Total Nr5a2
Esrrb
Nr0b1
Total Mbd3
EV-EV
Mbd3-EV
Klf4-EV
Klf4-Mbd3
Nr5a2-EV
Nr5a2-Mbd3
EpiSCs
iPSCs
H
Mbd3b MERKSPSGKKFRSKPQLARYLGGSMDLSTFDFRTGKMLMNKMNKSRQRVRYDSSNQVKGK 60
Mbd3c MARIWFGGWEISVP---DRPPATIQALAKHLPGPSNPPWTPVGAARCRVFSP-----QGK 52
Mbd3b PDLNTALPVRQTASIFKQPVTKITNHPSNKVKSDPQKAVDQPRQLFWEKKLSGLSAFDIA 120
Mbd3c PDLNTALPVRQTASIFKQPVTKITNHPSNKVKSDPQKAVDQPRQLFWEKKLSGLSAFDIA 112
Mbd3b EELVRTMDLPKGLQGVGPGCTDETLLSAIASALHTSTLPITGQLSAAVEKNPGVWLNTAQ 180
Mbd3c EELVRTMDLPKGLQGVGPGCTDETLLSAIASALHTSTLPITGQLSAAVEKNPGVWLNTAQ 172
Mbd3b PLCKAFMVTDDDIRKQEELVQQVRKRLEEALMADMLAHVEELARDGEAPLDKACAEEEEE 240
Mbd3c PLCKAFMVTDDDIRKQEELVQQVRKRLEEALMADMLAHVEELARDGEAPLDKACAEEEEE 232
Mbd3b EEEEEEEPEPERV- 253
Mbd3c EEEEEEEPEPERV- 245
I
2i/LIF
EV-EV
Nanog-EV
Mbd3c-EV
Nanog-Mbd3c
Oct4-GFP
500µm
AP
J
Total Nanog
Rex1
endOct4
Esrrb
Total Mbd3
Relative expression
EV-EV
Nanog-EV
Mbd3c-EV
Nanog-
Nanog-EV
Nanog-Mbd3c
preiPSCs (MKOS)
iPSCs (MKOS)
K
ESCs in 2i/LIF
+/+
fl/-
-/-
ex1fl/-
MBD3
a
b
c
αTUB
L
ESCs in 2i/LIF
+/+
ex1fl/-
Δex1/-
MBD3
a
b
c
αTUB
M
ex1fl/-
β-geo
+Cre
Δex1/-

Figure S4, related to Figure 4 - Overexpression of Mbd3/NuRD facilitates Nanog-mediated reprogramming.

(A) Phase and GFP images and AP staining of the iPSCs formed from preiPSCs overexpressing respective transgenes. It is to note that the expression level of Nanog transgene in the cell lines are similar (refer to Figure S4D). **(B)** Quantification of *Nanog*-GFP^{+} colonies in Figure S4A, after 12 days of 2i/LIF culture. Colony numbers are per 1.0 x 10^{5} preiPSCs. **(C)** qRT-PCR analysis of pluripotency-associated genes and *Mbd3* in transgenic preiPSCs used in Figures S4A-B, and corresponding derived iPSCs. **(D)** qRT-PCR analysis of NSC- and pluripotency-associated genes, and *Mbd3* in transgenic NSCs and corresponding derived iPSCs. **(E)** qRT-PCR analysis of retroviral transgenes, pluripotency-associated, and *Mbd3* in preiPSCs used in Figures 4E-H, and corresponding derived iPSCs. **(F)** qRT-PCR analysis of pluripotency-associated genes and *Mbd3* in transgenic EpiSCs and corresponding derived iPSCs. **(G)** qRT-PCR analysis of pluripotency-associated genes and *Mbd3* in EpiSCs transfected with *Klf4* or *Nr5a2*, and corresponding derived iPSCs. **(H)** ClustalW2 sequence alignment between Mbd3b isoform (cDNA used in this study, unless stated otherwise) and Mbd3c isoform (http://www.ebi.ac.uk/Tools/msa/clustalw2/). The isoforms differ in their N-terminus sequence, because of different translation start sites, Mbd3c being 8 amino acids shorter (dotted box). **(I)** Phase and GFP images and AP staining of the iPSCs formed from preiPSCs overexpressing respective transgenes. **(J)** qRT-PCR analysis of pluripotency-associated genes and *Mbd3* in transgenic preiPSCs and corresponding derived iPSCs. qRT-PCR values are normalized to *Gapdh* value and shown as relative to the highest value. The error bars indicate STDEV. **(J-L)** Western blot analysis of MBD3 and α-TUBULIN (αTUB) protein levels in ESC lines with different *Mbd3* genotypes cultured in 2i/LIF conditions: (K) *Mbd3*$^{+/+}$, *Mbd3*$^{fl/-}$, *Mbd3*$^{-/-}$ (derived from *Mbd3*$^{fl/-}$ after Cre-mediated deletion), and *Mbd3*$^{ex1fl/-}$; (L) *Mbd3*$^{+/+}$,

Mbd3$^{ex1fl/-}$ and *Mbd3*$^{\Delta ex1/-}$ (derived from *Mbd3*$^{ex1fl/-}$ after Cre-mediated deletion). Mbd3 isoforms a-c are indicated. **(M)** Schematic representation of *Mbd3* loci in *Mbd3*$^{ex1fl/-}$ and *Mbd3*$^{\Delta ex1/-}$ ESCs. Exons are indicated as dark grey boxes, non-coding sequences as unfilled boxes and light grey triangles represent loxP sites. *Mbd3* exon 1 deletion creates a hypomorphic MBD3 protein, with lower molecular weight.

Supplemental experimental procedures

Plasmids: pMX-Klf4, pMX-Oct4, pMX-c-Myc, pMX-Sox2, pMX-GFP and pLKO.1 (from Addgene repository); pPB-CAG-Nanog-pA-pgk-hph, pPB-CAG-DEST-pA-pgk-hph, pPB-CAG-Mbd3b-pA-pgk-bsd, pPB-CAG-Mbd3c-pA-pgk-bsd, pPB-CAG-Klf4-pA-pgk-hph, pPB-CAG-Nr5a2-pA-pgk-hph, pPB-CAG-DEST-pA-pgk-bsd, pPB-CAG-Klf2.2A.Nanog-Cherry-pA-pgk-zeo, pPB-CAG-Nr5a2-pA-pgk-hph, pCAG-Cre-ires-Puro and pCAG-Mbd3bihygro (made in the lab); pCAG-Cre-ERt2^{NLS}-IRES-BSD (from Joerg Betschinger); pPB-EOS-GFP-ires-Puro (pPB-EOS-GiP) (from Ge Guo); pCyL43 (PBase) and pPB-CAG-rtTA (from Sanger Institute's plasmid repository); PB-TAP IRI attP2LMKOSimO (O'Malley et al., 2013), PB-TAP IRI tetO-STEMCCAimO (Oct4-F2A-Klf4-ires-Sox2-E2A-c-Myc) and PB-TAP MKOSimO were generated by transferring STEMCCA reprogramming cassette into PB-TAP IRI piggyBac backbone with ires-mOrange (O'Malley et al., 2013; Sommer et al., 2009), and MKOSimO cassette into PB-tetO backbone (Woltjen et al., 2009), respectively. Sequences of the plasmids are available upon request.

Generation of transgenic cell lines: NSCs and preiPSCs were transfected using Amaxa Nucleofection Technology (Lonza AG). 2 x 10^6 cells were used per transfection. ESCs, iPSCs and EpiSCs were transfected in suspension using Lipofectamine 2000 (Invitrogen). Both protocols were performed according to manufacturer's instructions. PiggyBac transposon (pPB) plasmids were co-transfected with piggyBac transposase expression vector pBase (mixture of 1:1) to generate stable cell lines. Selection for transgenes was applied for at least three passages before setting up experiments.

siRNA knock-down: *Mbd3* knock-down was carried out using Flexitube siRNAs (Qiagen) against *Mbd3* (Table below). Four different siRNAs (Mm_Mbd3_1, 2, 3 and 5) were

individually tested and three of them were chosen to be used as a pool in subsequent experiments (Mm_Mbd3_1 - SI00206836, Mm_Mbd3_3 - SI00206850 and Mm_Mbd3_5 - SI02740045). AllStars Negative Control siRNA (1027280) was used as a control. The final concentration of siRNAs for transfection was 0.2 μM/cm^2. For reprogramming experiments, EpiSCs were transfected with the siRNAs using the Lipofectamine RNAiMAX reagent (Life Technologies) according to manufacturer's protocol. The media was switched to 2i/LIF 24h after transfection.

NSC and MEF reprogramming: To generate retroviruses encoding reprogramming factors, 2 x 10^6 PLAT-E cells (per transfection) were seeded in 10 cm dishes and transfected the next day with 9 μg of pMXs plasmids (OKM or OKMS in case of NSCs and MEFs, respectively; where indicated, pMXs-GFP was also used) using FuGENE 6 transfection reagent (Roche) according to manufacturer's instructions. The medium was switched to S+LIF the next day. The retrovirus-containing supernatants from PLAT-E cultures were collected 48 hours post-transfection and filtered using 0.45 μm filters. Polybrene was added to the filtered supernatants to a final concentration of 4 μg/ml. The mixture was then applied to the plated NSCs or MEFs. In case of NSCs, 24h after incubation, the virus-containing medium was replaced with Egf+Fgf2 medium for 2-3 days, after which the cells were switched to S+LIF medium to enable preiPSCs (reprogramming intermediates) formation. MEFs were maintained in S+LIF throughout. The emergent preiPSCs were then switched to 2i/LIF medium to induce complete reprogramming. Where indicated in the text, preiPSCs were passaged and transfected at the preiPSC stage, plated in S+LIF and switched to in 2i/LIF conditions 1-2 days later. *Nanog*-GFP MEF derived preiPSCs were chosen to address synergy between *Nanog* and *Mbd3* during reprogramming, since they convert to naïve pluripotency very inefficiently in 2i/LIF conditions, unless transfected with additional

factors (cells used in (Costa et al., 2013)). Where cells contained a reprogramming reporter (*Nanog*-GFPiresPuro or *Oct4*-GFPiresPuro), puromycin was added to 2i/LIF cultures six days after medium switch. Reprogramming experiments were ended 12 days after medium switch to 2i/LIF.

EpiSC reprogramming: Transgenic EpiSCs were plated in Fgf2/Act.A medium and switched to 2i/LIF conditions the next day. Once medium is switched to 2i/LIF EpiSCs no longer proliferate, unless they undergo reprogramming, making resulting iPSC colonies representative of initial plated EpiSC numbers. Where cells contained a reprogramming reporter (*Oct4*-GFPiresPuro of *EOS*-GFPiresPuro), 1 μg/ml puromycin was added to 2i/LIF cultures six days after medium switch. Reprogramming experiments were ended 12 days after medium switch to 2i/LIF. Number of EpiSCs plated differ from experiment to experiment and are indicated in the figure legends.

piggyBac transposon reprogramming: The PB-TAP IRI attP2LMKOSimO or PB-TAP IRI tetO-STEMCCAimO (500 ng), pPB-CAG-rtTA (500 ng) and pCyL43 piggyBac transposase expression vector (500 ng) were introduced into the MEFs with *Nanog-GFP* reporter (Chambers et al., 2007) seeded at 1 x 10^5 cells/wells in 6-well plates on the day before transfection using 6 μl of FugeneHD (Promega). Twenty-four hours later, culture medium was changed to S+LIF medium supplemented with 1.0 μg/ml doxycycline (dox), vitamin C (Sigma) (10 μg/ml) and Alk inhibitor A 83-01 (TOCRIS Bioscience) (500 nM) (+DVA). This medium was changed every two days until day 13 of reprogramming. Lentiviral infection using pLKO.1 (Addgene) with shRNA expression against *Mbd3* (shMbd3.2 and shMbd3.5), Hygromycin and Zeocin resistant genes (shHyg and shZeo) was carried out 24 hours after dox administration. Cell lysates from one of the triplicate at day 7 of reprogramming were used for Western blotting analysis to confirm MBD3 knockdown. For *Mbd3* exon1 deletion,

retroviral Cre-ERt2 expression vector was infected 24 hours after PB-TAP MKOSimO piggybac transfection with Fugene. At the same time culture medium was changed to S+LIF +DVA medium. 4-OHT was added at this point or 48 hours later, and kept in the culture medium for 48 hours. Cell lysates from one of the triplicate at day 7 of reprogramming were used for Western blotting analysis to confirm MBD3 depletion.

Embryoid Body differentiation: 1.5 x 10^6 cells were plated in non-adherent 10 cm bacterial dishes in serum minus LIF medium. Samples were collected at day 3, 5 and 7 of differentiation and analysed by qRT-PCR.

Blastocyst injection, chimera generation and germline transmission assessment: For blastocyst injection standard microinjection methodology using host blastocysts of C57BL/6 strain was employed. Floxed pPB transgenes were excised using TAT-Cre treatment before injection. Injected blastocysts were transferred to recipient mice to assess the contribution to chimeras. Generated chimeras were back-crossed with C57BL/6 mice to assess germline transmission.

Alkaline Phosphatase staining: Cells were fixed with a citrate-acetone-formaldehyde solution and stained for 30min using the Alkaline Phosphatase kit (Sigma-Aldrich) according to manufacturer's instructions.

Flow cytometry and imaging: Flow cytometry analysis was performed using an LSRFortessa analyser (BD). Cell sorting was performed using a MoFlo high-speed cell sorter. GFP was excited by a 488nm laser and detected using a 530/30 filter. All data analysis was performed using FlowJo software. Live cells were imaged with inverted Olympus IX51 microscope supplied with the Leica DFC310 FX digital colour camera, and processed with Leica software.

Western blotting: Protein extracts were obtained by incubation of cells in RIPA buffer (PBS, 1% NP40, 0.1% SDS, 0.1 mM EDTA supplemented with proteinase inhibitors, Roche). Total protein was quantified using BCA protein assay kit (Thermo Scientific) and the same amount of protein was loaded into gels (NuPAGE 10% Bis-Tris, Novex Life Technologies) for all the samples. Protein transfer was carried out using the iBlot system (iBlot Gel transfer stack nitrocellulose, Novex Life Technologies). Primary antibodies used: rabbit polyclonal MBD3 antibody (Bethyl, A302-528A, 1:2000), goat polyclonal MTA2 antibody (C-20) (Santa Cruz, sc-9447, 1:1000) and mouse monoclonal antibody α-TUBULIN (Abcam, ab7291, 1:5000). Secondary HRP-conjugated antibodies used: anti-rabbit (GE, NA934VS, 1:10000), anti-mouse (GE, NA931VS) and anti-goat (Santa Cruz, sc-2020, 1:2000). Blocking was carried out in 5% milk/0.1% tween in PBS (blocking solution) for 1h at RT. Primary antibodies were diluted in blocking solution and incubated with the membrane overnight at 4^0C. Three washing steps of 10 min were carried out with blocking solution after primary antibody incubation. Secondary antibodies were diluted in blocking solution and incubated with membranes for 1h at RT. Then three washing steps of 20min were performed with 0.1% tween in PBS. Membranes were developed using the ECL Prime detection kit (GE Healthcare) according to manufacturer's instructions. Membrane re-probing with another primary antibody was carried out after stripping the membrane with 100 mM β-Mercaptoethanol, 2% SDS and 62.5 mM Tris-HCl solution in water.

RNA isolation, cDNA synthesis and qRT-PCR: Total RNA was isolated from cells using the RNeasy mini kit (QIAGEN) in accordance with manufacturer's protocol. After purification, 1 µg of total RNA was reverse transcribed into cDNA using the SuperScript III First-Strand Synthesis SuperMix kit (Life Technologies). 10 ng of cDNA was used for qRT-PCR reactions that were set up in triplicates using either TaqMan Universal PCR Master Mix or

Fast SYBR Green Master Mix (Life Technologies). TaqMan gene expression assays (Applied Biosystems) or specific primers (Table below) were used for each gene analysed. qRT-PCR experiments were performed using StepOnePlus Real Time PCR System (Applied Biosystems). Delta Ct values with *Gapdh* were calculated and brought to power -2. Where indicated, the values were normalized to the highest value. Error bars represent ±S.D. of technical triplicates.

Cell proliferation analysis: Cell proliferation was assessed by counting cells every 24h using the Vi-Cell XR Cell Viability analyser (Beckman Coulter). For iPSCs proliferation analysis, 2000 cells were plated per 96-well plate and assayed with CellTiter 96 AQueous One Solution Cell Proliferation Assay (Promega) every 24h.

Primers and shRNA oligos used in the study.

***Mbd3* genotyping primers**	
Mbd3 gen. FP	ACTGCTCCAGCTTGGTACAG
Mbd3 gen. RP	AATCAGATCACTTCAGCTCC
**Primers used with SYBR green**	
Mbd3 FP	AGAAGAACCCTGGTGTGTGG
Mbd3 RV	TGTACCAGCTCCTCCTGCTT
Pl-1 FP	ATTTTGACTACCCTGCTTGGTCT
Pl-1 RP	TCTACATAACTGAGGAGGGGAAAG
GAPDH FP	CCCACTAACATCAAATGGGG
GAPDH RP	CCTTCCACAATGCCAAAGTT
Olig2 FP	CTGCTGGCGCGAAACTACAT
Olig2 RP	CGCTCACCAGTCGCTTCAT
Blbp FP	AGACCCGAGTTCCTCCAGTT
Blbp RP	ATCACCACTTTGCCACCTTC
Sox2 FP	TCCAAAAACTAATCACAACAATCG
Sox2 RP	GAAGTGCAATTGGGATGAAAA
Nr5a2 FP	CCAGAAAACATGCAAGTGTCTCAA
Nr5a2 RP	CGTGAGGAGACCGTAATGGTA

**Applied Biosystems Taqman probes**	
Nanog	Mm02384862_g1
Rex1	Mm03053975_g1
Klf4	Mm00516104_m1
Klf2	Mm01244979_g1
Fgf5	Mm00438919_m1
Lefty1	Mm00438615_m1
T-Brachyury	Mm01318252_m1
GAPDH	4352339E
Esrrβ	Mm00442411_m1
Gata4	Mm00484689_m1
Nr0b1	Mm00431729_m1
**Applied Biosystems custom Taqman probes**	
Retroviral Klf4 FP	TGGTACGGGAAATCACAAGTTTGTA
Retroviral Klf4 RP	GAGCAGAGCGTCGCTGA
Retroviral Klf4 probe	FAM-CCCCTTCACCATGGCTG-MGB
Retroviral Oct4 FP	TGGTACGGGAAATCACAAGTTTGTA
Retroviral Oct4 RP	GGTGAGAAGGCGAAGTCTGAAG
Retroviral Oct4 probe	FAM-CACCTTCCCCATGGCTG-MGB
Retroviral cMyc FP	TGGTACGGGAAATCACAAGTTTGTA
Retroviral cMyc RP	GGTCATAGTTCCTGTTGGTGAAGTT
Retroviral cMyc probe	FAM-CCCTTCACCATGCCCC-MGB
Retroviral Sox2 FP	TGGTACGGGAAATCACAAGTTTGTA
Retroviral Sox2 RP	GCCCGGCGGCTTCA
Retroviral Sox2 probe	FAM-CTCCGTCTCCATCATGTTAT-MGB
Endogenous Oct4 FP	TTCCACCAGGCCCCC
Endogenous Oct4 RP	GGTGAGAAGGCGAAGTCTGAAG
Endogenous Oct4 probe	FAM-CCCACCTTCCCCATGGCT-MGB
**siRNAs from Qiagen**	
Mm_Mbd3_1 - SI00206836	CGGAAAGATGTTGATGAACAA
Mm_Mbd3_2 - SI00206843	ACCGGTGACCAAGATCACCAA

Mm_Mbd3_3 - SI00206850		CAGGACCATGGACTTGCCCAA
Mm_Mbd3_5 - SI02740045		AAGTCACTTTCCTTCAATAAA
AllStars Negative Control siRNA		Cat. No.: 1027280
**Upper strand oligos used for lentiviral knockdown vectors**		
shMbd3.2	Upper strand	CCGGGCGCTATGATTCTTCCAACCACTCGAGTGGTTGGAAGAATCATAGCGCTTTTT
	Bottom strand	AATTAAAAAGCGCTATGATTCTTCCAACCACTCGAGTGGTTGGAAGAATCATAGCGC
shMbd3.5	Upper strand	CCGGAAGTCACTTTCCTTCAATAAACTCGAGTTTATTGAAGGAAAGTGACTTTTTTT
	Bottom strand	AATTAAAAAAAGTCACTTTCCTTCAATAAACTCGAGTTTATTGAAGGAAAGTGACTT
shHyg	Upper strand	CCGGGCGAAGAATCTCGTGCTTTCACTCGAGTGAAAGCACGAGATTCTTCGCTTTTT
	Bottom strand	AATTAAAAAGCGAAGAATCTCGTGCTTTCACTCGAGTGAAAGCACGAGATTCTTCGC
shZeo	Upper strand	CCGGGCCAAGTTGACCAGTGCCGTTCTCGAGAACGGCACTGGTCAACTTGGCTTTTT
	Bottom strand	AATTAAAAAGCCAAGTTGACCAGTGCCGTTCTCGAGAACGGCACTGGTCAACTTGGC

Supplemental References

Chambers, I., Silva, J., Colby, D., Nichols, J., Nijmeijer, B., Robertson, M., Vrana, J., Jones, K., Grotewold, L., and Smith, A. (2007). Nanog safeguards pluripotency and mediates germline development. Nature 450, 1230-U1238.

Costa, Y., Ding, J., Theunissen, T.W., Faiola, F., Hore, T.A., Shliaha, P.V., Fidalgo, M., Saunders, A., Lawrence, M., Dietmann, S., et al. (2013). NANOG-dependent function of TET1 and TET2 in establishment of pluripotency. Nature 495, 370-374.

O'Malley, J., Skylaki, S., Iwabuchi, K.A., Chantzoura, E., Ruetz, T., Johnsson, A., Tomlinson, S.R., Linnarsson, S., and Kaji, K. (2013). High-resolution analysis with novel cell-surface markers identifies routes to iPS cells. Nature 499, 88-91.

Sommer, C.A., Stadtfeld, M., Murphy, G.J., Hochedlinger, K., Kotton, D.N., and Mostoslavsky, G. (2009). Induced pluripotent stem cell generation using a single lentiviral stem cell cassette. Stem Cells 27, 543-549.

Woltjen, K., Michael, I.P., Mohseni, P., Desai, R., Mileikovsky, M., Hamalainen, R., Cowling, R., Wang, W., Liu, P.T., Gertsenstein, M., et al. (2009). piggyBac transposition reprograms fibroblasts to induced pluripotent stem cells. Nature 458, 766-U106.
